# Prion replication environment defines the fate of prion strain adaptation

**DOI:** 10.1371/journal.ppat.1007093

**Published:** 2018-06-21

**Authors:** Elizaveta Katorcha, Nuria Gonzalez-Montalban, Natallia Makarava, Gabor G. Kovacs, Ilia V. Baskakov

**Affiliations:** 1 Center for Biomedical Engineering and Technology, University of Maryland School of Medicine, Baltimore, Maryland, United States of America; 2 Department of Anatomy and Neurobiology, University of Maryland School of Medicine, Baltimore, Maryland, United States of America; 3 Institute of Neurology, Medical University of Vienna, Vienna, Austria; Dartmouth Medical School, USA, UNITED STATES

## Abstract

The main risk of emergence of prion diseases in humans is associated with a cross-species transmission of prions of zoonotic origin. Prion transmission between species is regulated by a species barrier. Successful cross-species transmission is often accompanied by strain adaptation and result in stable changes of strain-specific disease phenotype. Amino acid sequences of host PrP^C^ and donor PrP^Sc^ as well as strain-specific structure of PrP^Sc^ are believed to be the main factors that control species barrier and strain adaptation. Yet, despite our knowledge of the primary structures of mammalian prions, predicting the fate of prion strain adaptation is very difficult if possible at all. The current study asked the question whether changes in cofactor environment affect the fate of prions adaptation. To address this question, hamster strain 263K was propagated under normal or RNA-depleted conditions using serial Protein Misfolding Cyclic Amplification (PMCA) conducted first in mouse and then hamster substrates. We found that 263K propagated under normal conditions in mouse and then hamster substrates induced the disease phenotype similar to the original 263K. Surprisingly, 263K that propagated first in RNA-depleted mouse substrate and then normal hamster substrate produced a new disease phenotype upon serial transmission. Moreover, 263K that propagated in RNA-depleted mouse and then RNA-depleted hamster substrates failed to induce clinical diseases for three serial passages despite a gradual increase of PrP^Sc^ in animals. To summarize, depletion of RNA in prion replication reactions changed the rate of strain adaptation and the disease phenotype upon subsequent serial passaging of PMCA-derived materials in animals. The current studies suggest that replication environment plays an important role in determining the fate of prion strain adaptation.

## Author summary

The main risk of emergence of prion diseases in humans is associated with a cross-species transmission of prions of zoonotic origin. Prion transmission between species is regulated by a species barrier. Amino acid sequences of host prion protein and donor prions are believed to be the main factors that control species barrier and strain adaptation. Yet, despite our knowledge of the primary structures of mammalian prions, predicting the fate of prion strain adaptation is very difficult. The current study asked the question whether changes in cofactor environment affect the fate of prions adaptation. To address this question, hamster prion strain was propagated under normal or RNA-depleted conditions *in vitro* first using mouse and then hamster substrates. This work demonstrated that depletion of RNA in prion replication reactions changed the rate of strain adaptation and the disease phenotype upon subsequent serial passaging in animals. The current studies suggest that replication environment plays an important role in determining the fate of prion strain adaptation.

## Introduction

Prion diseases are a group of fatal neurodegenerative diseases of humans and other mammals that can arise spontaneously or via transmission [1]. The transmissible agent of prion disease consists of a prion protein in β-sheet rich self-propagating states referred to as PrP^Sc^ that template conversion of the same protein in its normal, cellular state (PrP^C^) into disease-related pathogenic state [2–6].

The main risk of emergence of prion diseases in humans is associated with a cross-species transmission of prions of zoonotic origin. Transmission of prions or PrP^Sc^ between species is less efficient in comparison to transmissions within the same species due to the species barrier (reviewed in [7]). According to a traditional view, the species barrier arises because of differences between amino acid sequences of donor PrP^Sc^ and host PrP^C^, whereas the magnitude of a barrier is believed to be determined by the extent to which the strain-specific conformation of donor PrP^Sc^ can be accommodated within the primary structure of the new host PrP [8–10]. While the role of amino acid sequences in regulating the species barrier has been supported by numerous studies [7,8,11–13], multiple exceptions have been described over the years, arguing that other yet unknown factors contribute to the barrier. For instance, in certain lines of transgenic mice expressing human PrP^C^ the transmission of a new variant Creutzfeldt-Jakob Disease occurred at full attack rate. Yet in other lines of humanized mice the transmission showed significant barrier as judged from long incubation times, incomplete attack rates or lack of clinical diseases despite identity in amino acid sequences of host PrP^C^ and donor PrP^Sc^ [14,15]. Moreover, several studies illustrated that prions from a variety of species could be transmitted very effectively to the bank vole despite differences in amino acid sequences showing very little if any species barrier [16–18]. These studies suggest that the bank vole is a universal host.

Successful cross-species prion transmission is typically accompanied by strain adaptation that might result in stable changes of strain-specific disease phenotype, a phenomenon known as prion strain mutation. Prion strain mutation has been attributed to changes in PrP^Sc^ conformation that occur as a result of adaptation to a new host [19,20]. Despite the fact that amino acid sequences of the prion protein are known for many mammalian species, predicting the fate of prion strain adaptation upon cross-species transmission is very difficult if possible at all. A comprehensive mechanism explaining species barrier has yet to be developed.

A number of studies in the last decade provided convincing evidence that cellular molecules of non-protein nature including RNAs and lipids assist prion replication [4,5,21–25]. *In vitro* studies of prion replication using Protein Misfolding Cyclic Amplification (PMCA) suggested that RNAs and polyanions form favorable biochemical environment that assists replication [4,21]. However, the mechanism by which non-protein co-factors assist prion conversion is not well defined [26–29]. Moreover, the extent to which non-protein co-factors specify strain-specific properties is not clear [23]. Interestingly, the effects of RNAs in facilitating replication of prion were found to be species- and strain-dependent [25,30,31]. These studies suggested that optimal prion replication might require species- and strain-specific cofactors. Bearing this in mind, we hypothesized that cofactor environment might play an important role in defining the fate of prion strain adaptation.

Previously, we showed that a change in co-factor environment alone, and specifically reversible depletion of total cellular RNA, without changes in PrP primary sequence led to stable changes in strain-specific physical features of PrP^Sc^ [32]. In the current study, we asked the questions whether the changes in prion replication environment affect the fate of prions adaptation. To address this question, hamster strain 263K was propagated in normal or RNA-depleted conditions in serial PMCA with beads (PMCAb) in mouse brain homogenate, and then re-adapted back to hamster brain homogenate under normal or RNA-depleted conditions. Subsequent serial transmission revealed that PMCAb-derived products formed under RNA-depleted and normal conditions induced different disease phenotypes in animals.

## Results

**Fig 1:**
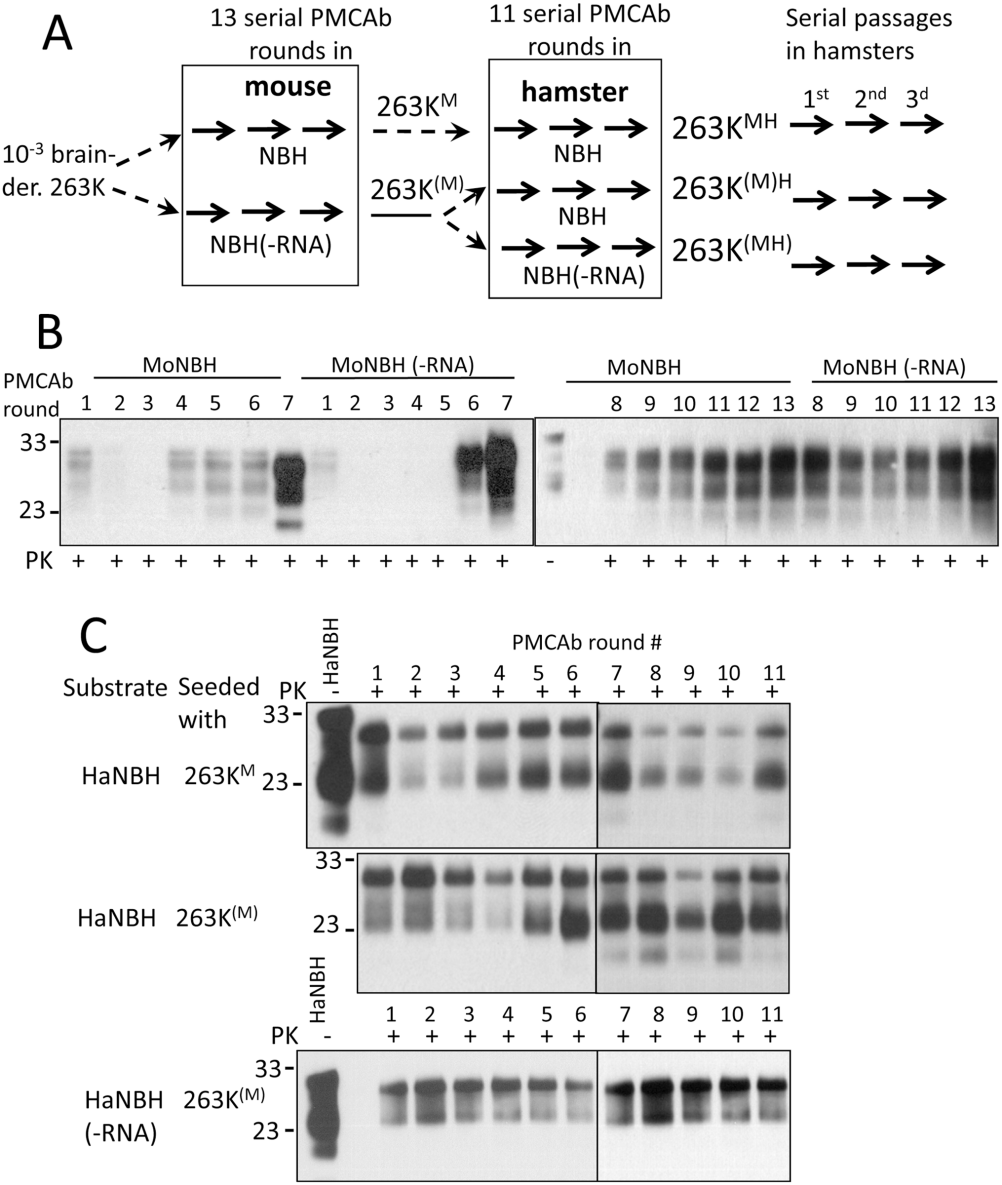
Amplification of 263K in serial PMCA under RNA-depleted or normal conditions. (A) Schematic diagram illustrating experimental design. (B) Adaptation of 263K to mouse substrate. Serial PMCAb reactions were seeded with 10^3^-diluted 263K brain material and conducted under normal or RNA-depleted conditions (-RNA) using mouse brain homogenates (MoNBH) as a substrate for thirteen rounds with 10-fold dilutions between rounds. (C) Re-adaptation of PMCAb products to hamster substrate. Serial PMCAb reactions were seeded with 263K^M^ or 263K^(M)^ PMCAb-derived materials and conducted under normal or RNA-depleted conditions (-RNA) using hamster brain homogenates (HaNBH) as a substrate for eleven rounds with 10-fold dilutions between rounds. All PMCAb-derived products were treated with 50 μg/ml PK. Western blots were stained with D18 antibody (epitope 132–156).

For testing whether the fate of prion adaptation depends on cofactor environment, the following experiments were conducted (Fig 1A). First, hamster strain 263K was propagated in serial PMCAb for thirteen rounds under normal or RNA-depleted conditions using mouse brain homogenate as a substrate (Fig 1A). RNA depletion in brain homogenate was confirmed by an agarose gel (S1 Fig). The products of PMCAb reactions in normal and RNA-depleted mouse brain homogenates will be referred to as 263K^M^ and 263K^(M)^, respectively. Second, 263K^(M)^ were readapted to hamster substrate by propagating in serial PMCAb reactions under normal or RNA-depleted conditions using hamster brain homogenates. The PMCAb products generated in normal and RNA-depleted hamster brain homogenates will be referred to as 263K^(M)H^ and 263K^(MH)^, respectively (Fig 1A). In parallel, 263K^M^ was readapted to hamster substrate in serial PMCAb reactions under normal conditions. The products of this reaction will be referred to as 263K^MH^ (Fig 1A).

Analysis of PK-resistant PMCAb products by Western blotting revealed that propagation of 263K in mouse substrate displayed a phenomenon similar to the transmission barrier (Fig 1B). This result was consistent with the previous studies on adaptation of hamster strains to mouse substrate in PMCA [33,34]. Amplification of 263K in a mouse substrate was observed regardless of presence of RNA in PMCAb (Fig 1B). Upon re-adaptation to hamster substrate, both 263K^M^ and 263K^(M)^ displayed relatively stable replication in hamster brain homogenate without significant barrier suggesting that 263K^M^ and 263K^(M)^ contain conformations compatible with hamster substrate. (Fig 1C). In addition to PK-resistant products of standard size, shorter PK-resistant band of approximately 23 kDa was visible in all three reactions.

**Fig 2:**
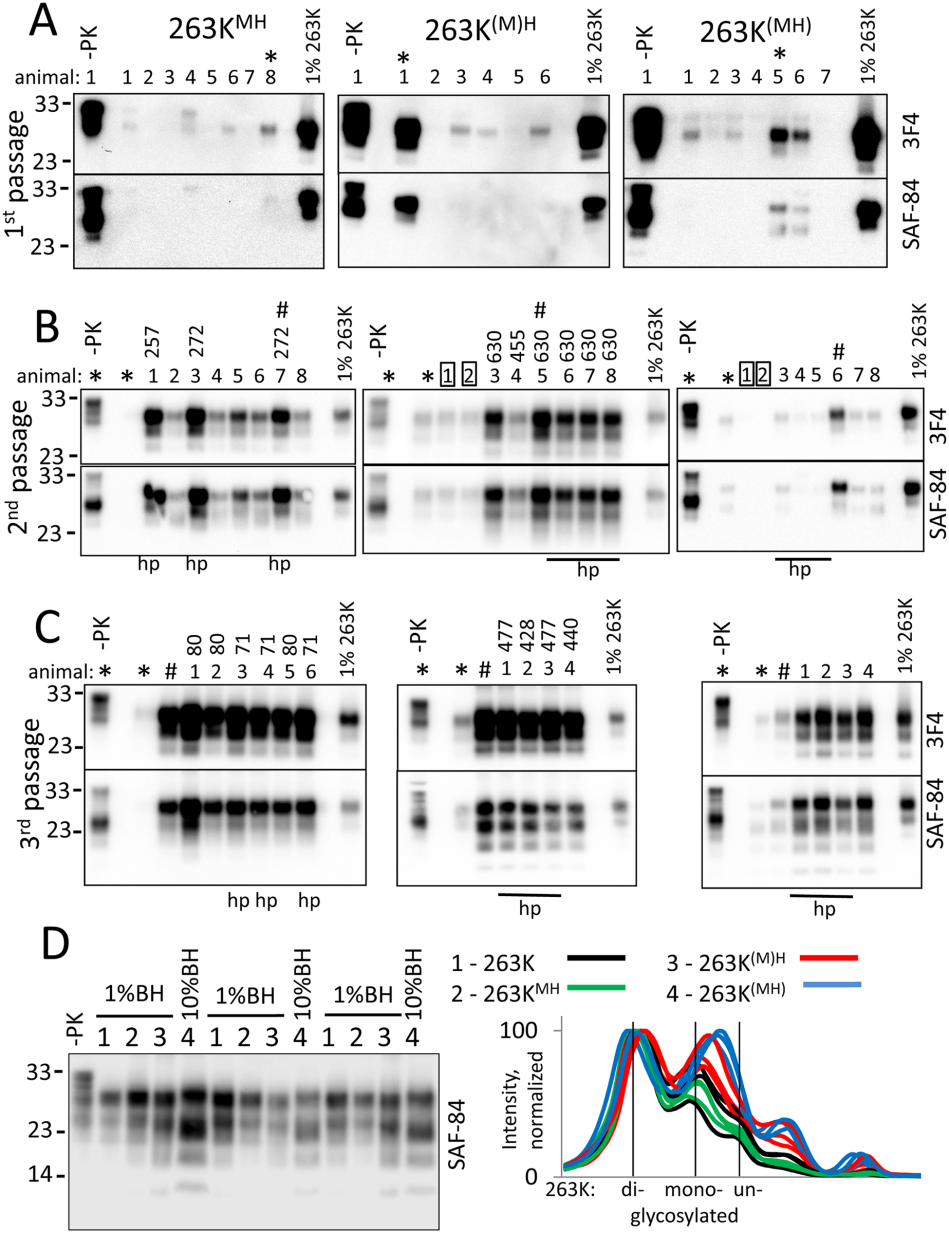
Serial passaging of PMCAb-derived 263K^MH^, 263K^(M)H^ and 263K^(MH)^ in Syrian hamsters. (A) Western blot analysis of brain material from hamsters inoculated with PMCAb-derived 263K^MH^, 263K^(M)H^ or 263K^(MH)^. Western blots were overexposed for detecting minor amounts of PK-resistant material. Brains marked by asterisks were used to prepare inocula for the second passage. (B) Western blot analysis of brain material from the animals of the 2^nd^ passage of 263K^MH^, 263K^(M)H^ or 263K^(MH)^. Brain materials used to prepare inocula for the second and third passages are marked by asterisks and hash signs, respectively. Rectangles show animals in 263K^(M)H^ and 263K^(MH)^ groups euthanized at 347 days postinoculation. (C) Western blot analysis of brain material from the animals of the 3^rd^ passage of 263K^MH^, 263K^(M)H^ or 263K^(MH)^. Brain materials used to prepare inocula for the second and third passages are marked by asterisks and hash signs, respectively, and are shown as references. In all panels, Western blots were stained with 3F4 (top panels) or SAF-84 antibody (bottom panels). 1% 263K brain homogenate was used as a cross-reference between panels. Brains used for histopathology analysis are marked by “hp”. For the animals that developed clinical diseases, the incubation times to the terminal stage are indicated. (D) Comparison of glycoform composition and molecular weight of PK-resistant products in brain material from the animals of 263K group (#1, black lines), the 3^rd^ passages of 263K^MH^ (#2, green lines), 263K^(M)H^ (#3, red lines) or 263K^(MH)^ (#4, blue lines) by Western blot (left panel); n = 3 individual animals per group. For 263K, 263K^MH^ and 263K^(M)H^ 1% brain homogenates (BH) were loaded onto SDS-PAGE, whereas 10% brain homogenates were loaded for 263K^(MH)^. Right panel shows intensity profiles for 263K, 263K^MH^, 263K^(M)H^ and 263K^(MH)^ group normalized by the intensity of diglycosylated isoform within individual samples. Vertical black lines identify positions of di-, mono- and unglycosylated isoforms for 263K group. With the exception of the brain materials shown on the first lanes (-PK), all brain materials were treated with 20 μg/ml PK.

To test whether RNA depletion changed the fate of prion adaptation, Syrian hamsters were inoculated with PMCAb-derived 263K^MH^, 263K^(M)H^ or 263K^(MH)^. By the end of PMCAb experiments, the original 263K brain material was diluted 10^27^-fold in PMCAb-derived 263K^MH^, 263K^(M)H^ and 263K^(MH)^; this dilution is approximately 10^17^ fold higher than the limiting dilution of 263K [35]. None of the animals from three groups developed any notable signs of prion disease for up to 518 days post inoculation (Table 1). Analysis of their brains by Western blotting revealed relatively minor yet variable amounts of PK-resistant material in all three groups (Fig 2A).

**Table 1:** Serial transmission of PMCAb-derived material in Golden Syrian hamsters.

Inoculum	1^st^ passage	2^nd^ passage		3^rd^ passage		
	n_sick_/n_t_	n_sick_/n_t_	inc time ^d^, dpi	n_sick_/n_t_	inc time ^d^, dpi	euthanized, dpi
263K^MH^	0/8 ^a^	3/8 ^b^	3 at 246	6/6	2 at 44, 52, 3 at 66	3 at 71, 3 at 80
263K^(M)H^	0/6 ^a^	0/2 ^c^ +6/6	2 at 392, 400, 463, 2 at 575	4/4	2 at 274, 2 at 365	428, 440, 2 at 477
263K^(MH)^	0/7 ^a^	0/2 ^c^ +0/6 ^b^	n/a	0/4	n/a	614

^a^ The animals from the 1^st^ passage were euthanized at 518 days postinoculation

^b^ The animals from these groups that did not develop disease were euthanized at 503 days postinoculation.

^c^ Two animals were euthanized at 347 days postinoculation in the absence of clinical signs.

^d^ The incubation time to first clinical signs is shown.

Animals with the highest amounts of PK-resistant material from each group were selected for the 2^nd^ passage. Out of the eight animals from the 2^nd^ passage of 263K^MH^, three developed clinical signs of prion disease at 246 days postinoculation and where euthanized at the terminal stage a few days later, 257 or 272 days postinoculation (Table 1). Animals that developed the disease showed considerably more PrP^Sc^ relative to the animals from the same group that were euthanized in the absence of the disease at 503 days postinoculation (Fig 2B). To probe the pace of adaptation in other two groups, two animals from each the 263K^(M)H^ and 263K^(MH)^ groups were euthanized at 347 days postinoculation in the absence of clinical signs (Fig 2B). Notably, by 347 days, the signal intensity in 263K^(M)H^ groups was the same as in the corresponding inoculum, whereas in the 263K^(MH)^ group the signal dropped considerably lower relative to the signal in corresponding inoculum (Fig 2B). This result suggests a low replication and/or a high clearance rate in the 263K^(MH)^ group relative to the other two groups. Nevertheless, all remaining six animals from the 2^nd^ passage of 263K^(M)H^ developed clinical signs after incubation time ranging from 392 to 575 days postinoculation (Table 1). In this group, the clinical signs progressed much more slowly in comparison to the animals from the 2^nd^ passage of 263K^MH^ (Table 1). Large amounts of PrP^Sc^ were found in all clinical animals from 263K^(M)H^ group (Fig 2B). None of the animals from the 2^nd^ passage of 263K^(MH)^ developed clinical diseases for up to 503 days postinoculation (Table 1). Lack of disease in this group correlated well with very minor amounts of PK-resistant material observed in their brain (Fig 2B).

**Fig 3:**
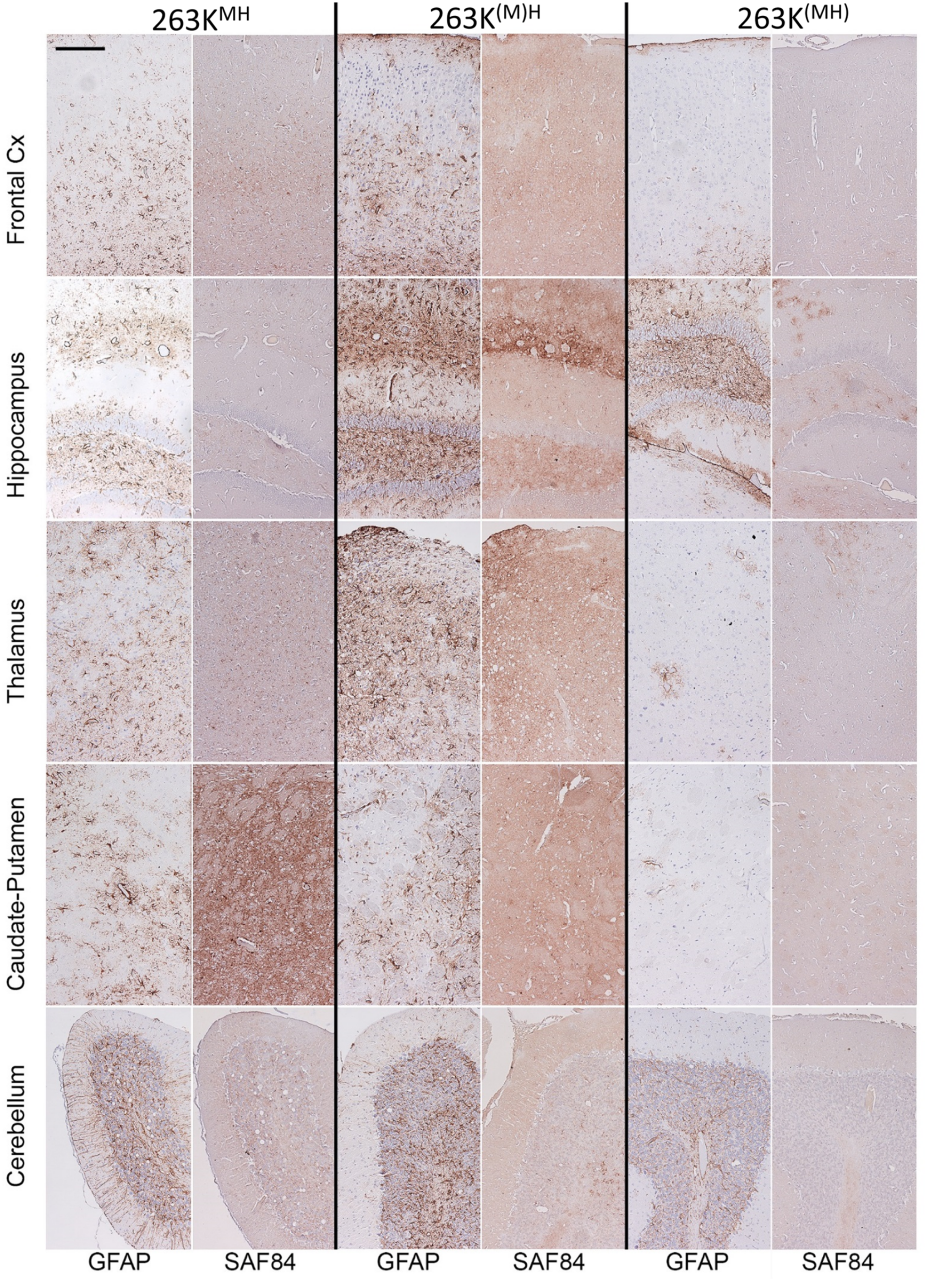
Histopathological analysis of brains from the 2^nd^ passage of 263K^MH^, 263K^(M)H^ or 263K^(MH)^. Representative images of the frontal cortex, hippocampus, thalamus, caudate-putamen, or cerebellum stained with anti-PrP SAF-84 or anti-GFAP antibodies as indicated. Scale bar = 100 μm for all images.

Three animals from each group of the 2^nd^ passage were analyzed using histopathology for deposition of PrP^Sc^, reactive astrogliosis and vacuolation. From the 263K^MH^ and 263K^(M)H^ groups, only animals that developed clinical disease were selected for histopathological analysis. Consistent with lack of clinical signs and low amounts of PrP^Sc^ observed by Western blots, 263K^(MH)^ animals exhibited minor vacuolation and astrogliosis, and only focal deposits of PrP mostly in the thalamus and hippocampus (Fig 3, S2 and S3 Figs). In contrast to the 263K^(MH)^ group, animals from 263K^MH^ and 263K^(M)H^ groups displayed more substantial deposition of disease-associated PrP and more pronounced astrogliosis with respect to anatomical distribution and degree of changes (Fig 3 and S3 Fig). Disease-associated PrP was found in form of diffuse/synaptic fine deposits, occasional mini-plaques, and less frequent pericellular PrP deposits. In periventricular areas amorphous deposits were seen. Furthermore, subependymal amorphous plaque-like deposits were noted in 263K^MH^ and 263K^(M)H^ groups, but were very mild in 263K^(MH)^ group. Nevertheless, as judged from the lesions and PrP deposition scored across brain regions, 263K^(M)H^ and 263K^(MH)^ groups displayed similar profile shapes for both the lesion and PrP immunoreactivity, albeit the 263K^(MH)^ group was less affected than 263K^(M)H^ group (S3 Fig). The shape of PrP immunoreactivity profile of 263K^MH^ group was notably different when compared to the profiles of 263K^(M)H^ and 263K^(MH)^ suggesting that different strains might be emerging in 263K^MH^ and 263K^(M)H^ / 263K^(MH)^ groups (S3 Fig). Animals from the age-matched control group lacked any deposition of PrP^Sc^ or spongiform degeneration (S4 Fig).

For the 3^rd^ passage, one brain with the strongest signal on Western blot was selected from each group. All animals from the 263K^MH^ passage showed clinical signs similar to those of 263K between 44 and 66 days postinoculation (Table 1). In this group, the disease progressed quickly and all animals were euthanized at 71 or 80 days postinoculation. In comparison to the 263K^MH^ group, the animals from the 3^rd^ passage of 263K^(M)H^ showed the first clinical signs after significantly longer incubation time (Table 1). Animals of this group developed an agitated, fidgeting behavior, dry skin, rough and patchy coat. The disease progressed slower in comparison to the 263K^MH^ group and animals were euthanized at the terminal stages between 428 and 477 days postinoculation. Surprisingly, none of the animals from the 3^rd^ passage of 263K^(MH)^ developed clinical signs for up to 614 days postinoculation (Table 1). Interestingly, in both 263K^MH^ and 263K^(M)H^ groups, the intensities of PrP^Sc^ signal on Western blot were very similar to the intensities of corresponding brain materials from the 2^nd^ passage used for inoculations (Fig 2C). The 263K^(MH)^ group showed lower amounts of PrP^Sc^ in comparison to the 263K^MH^ and 263K^(M)H^ groups, yet substantial increase was observed in the 3^rd^ passage of 263K^(MH)^ relative to the animals from the 2^nd^ passage of 263K^(MH)^ (Fig 2C).

A detailed comparison of the PK-resistance profiles by Western blot revealed that the relative proportions of the mono- and unglycosylated isoforms are higher in 263K^(M)H^ and 263K^(MH)^ groups relative to the 263K^MH^ or 263K groups (Fig 2D). In addition, 263K^(M)H^ and 263K^(MH)^ groups showed slight shifts in the mobility of the PK-resistant products toward the lower molecular weight (Fig 2D). Moreover, both 263K^(M)H^ and 263K^(MH)^ groups showed an additional lower molecular weight PK-resistant product at ~12 kDa (Fig 2C and 2D). Appearance of the lower molecular weight bands under non-denaturing conditions suggests that PrP^Sc^ might expose internal PK-cleavage sites in the central region of PrP as reported previously [36], or that alternative PK-resistant states might exist in 263K^(M)H^ and 263K^(MH)^ groups.

**Fig 4:**
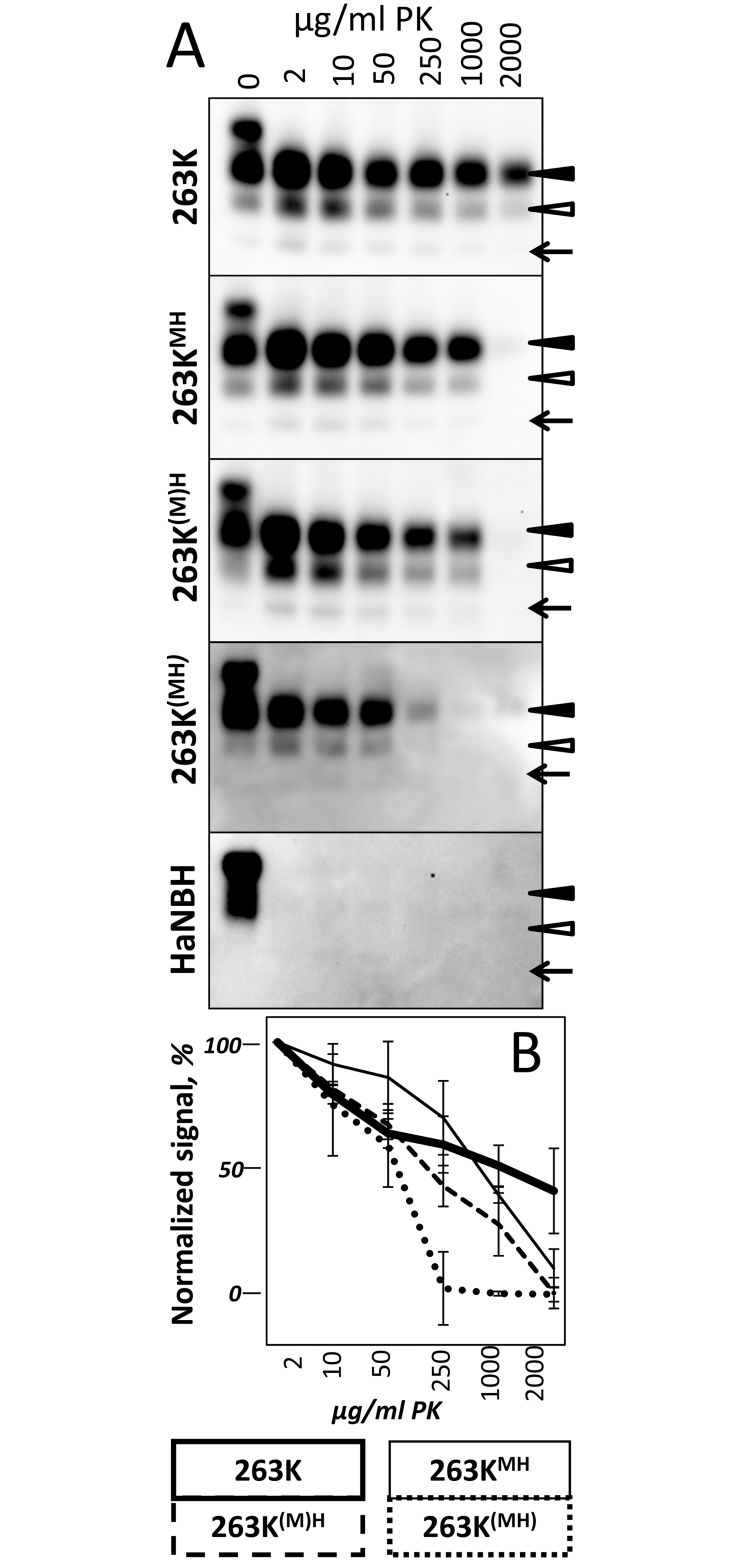
Analysis of the proteolytic resistance of PrP^Sc^ in animals from the 3^rd^ passage of 263K^MH^, 263K^(M)H^ and 263K^(MH)^. (A) Representative Western blots of brain materials from 263K^MH^, 263K^(M)H^ and 263K^(MH)^ groups treated with increasing concentrations of PK as indicated. PK treatment of 263K brain material and 10% normal brain control are shown as references. Black and white triangles mark di- and monoglycosylated glycoforms, respectively, whereas arrows mark the unglycosylated form. (B) PK-resistance profile of brain materials from 263K^MH^, 263K^(M)H^ and 263K^(MH)^ groups, and 263K brain material. The data were normalized relative to the intensity of the PK-resistant bands at 2 μg/ml PK. Means ± SD (n = 3 individual animals per group). Western blots were stained with 3F4 antibody.

**Fig 5:**
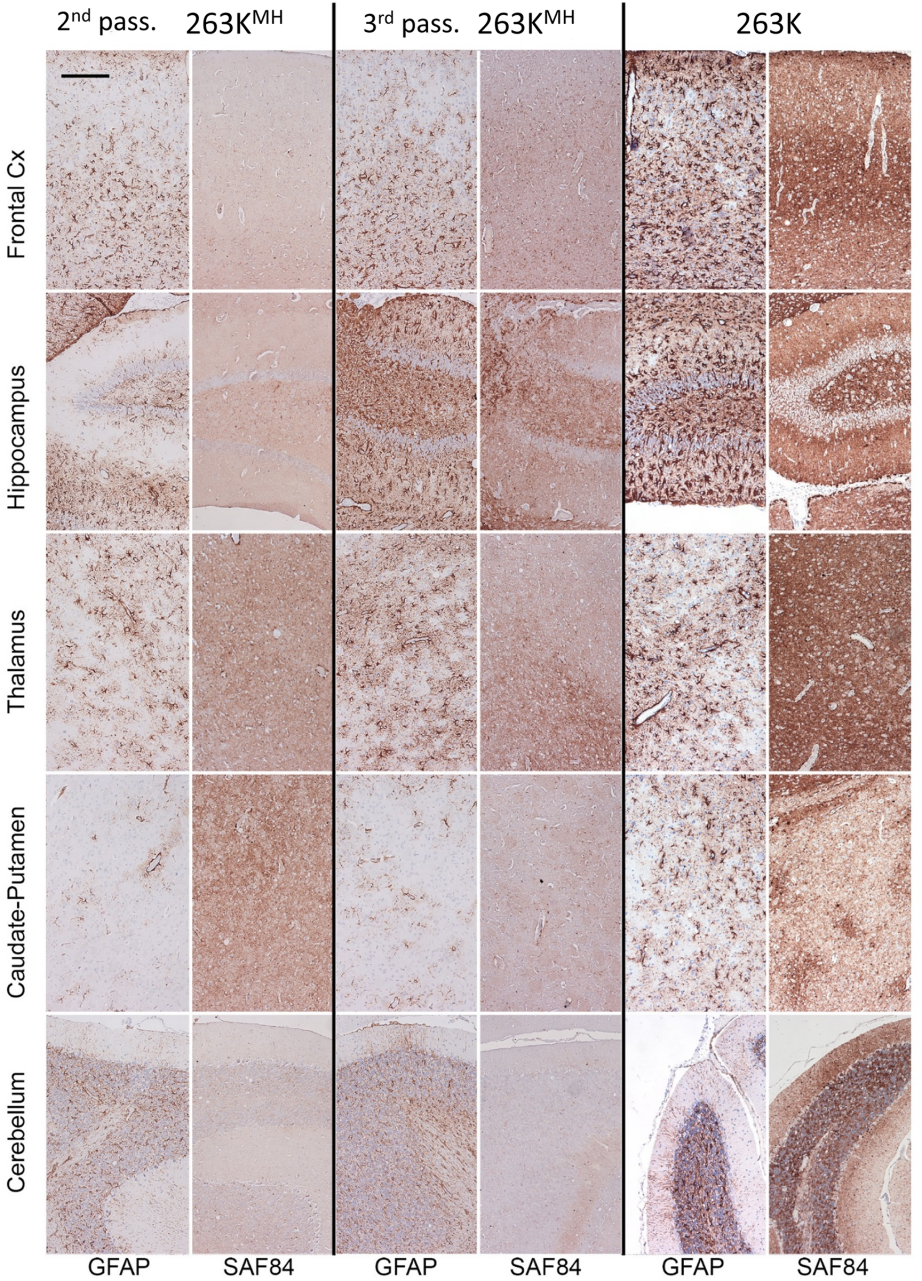
Comparative histopathological analysis of animals from the 2^nd^ and 3^rd^ passages of 263K^MH^ and animals inoculated with 263K. Representative images of the frontal cortex, hippocampus, thalamus, caudate-putamen, or cerebellum stained with anti-PrP SAF-84 or anti-GFAP antibodies as indicated. Depending on the brain area, PrP deposition and reactive gliosis were very mild or lacking in the 263K^(MH)^ group. Scale bar = 100 μm for all images.

To probe further the differences in physical properties of PrP^Sc^, brain materials from four groups were analyzed using two assays: (i) treated with increasing concentrations of PK and (ii) subjected to increasing concentrations of GdnHCl following by PK treatment. The PK-resistance profiles were found to be similar for 263K^MH^, 263K^(M)H^ and 263K groups (Fig 4A and 4B). In 263K^(MH)^ group, PrP^Sc^ was considerably more sensitive to digestion at high concentrations of PK (above 50 μg/ml) relative to PrP^Sc^ from other three groups (Fig 4A and 4B). In the experiments on denaturation, PrP^Sc^ of 263K^MH^ and 263K groups exhibited very similar GdnHCl-induced denaturation profiles (S5 Fig). A shift of the PK-resistant products toward lower molecular weight bands was observed in both groups at 3 M GdnHCl, an indication that internal PK-cleavage sites have been exposed in PrP^Sc^ upon denaturation (S5 Fig). The GdnHCl-induced denaturation profiles of 263K^(M)H^ and 263K^(MH)^ groups were notably different from those of the 263K^MH^ and 263K groups as well as from each other (S5 Fig). In the 263K^(M)H^ group, the lower molecular weight bands were barely visible at the low concentrations of GdnHCl, but increased gradually after 2M of GdnHCl (S5 Fig). In the 263K^(MH)^ groups, the low molecular weight bands were visible well at the low concentrations of GdnHCl; their intensity decreased in parallel with the drop of intensity of standard PK resistant bands at high concentrations of GdnHCl. In summary, 263K^MH^, 263K^(M)H^ and 263K^(MH)^ groups displayed three different patterns of GdnHCl-induced denaturation, with the pattern of 263K^MH^ being very similar to that of 263K. Comparative analysis of animals from the 2^nd^ and 3^rd^ passages of 263K^MH^ and the original 263K revealed similar histopathological features with respect to spongiform degeneration, reactive astrogliosis or PrP immunoreactivity in all three groups (Fig 5, S6 Fig). In all three groups, immunostaining for PrP displayed the following types of PrP deposits: diffuse/synaptic fine deposits, intra- and perineuronal deposits, plaques and mini-plaques in the subependymal area, small granular accumulations on the ependyma, and amorphous plaque-like deposits in the subpial and subependymal regions (Fig 5). These PrP deposits can be seen under large magnification in S7 Fig. With respect to anatomical distribution, the pathology in the 2^nd^ and 3^rd^ passages of 263K^MH^ was reminiscent yet not identical to that of scrapie 263K (Fig 5, S8 Fig). While the intensity of the PrP deposition in the 3^rd^ passage increased in comparison to the 2^nd^ passage, it did not reach the intensity observed for 263K group in a few brain regions such as the cerebellum and caudate-putamen (Fig 5, S8 Fig). The intensity of reactive astrogliosis correlated well with the intensity of PrP deposition and its anatomical distribution in all three groups (Fig 5). Again, the reactive astrogliosis was more prominent in the animals of the 3^rd^ passage relative to the 2^nd^ passage of 263K^MH^. Overall, the histopathological analysis confirmed that the disease phenotype of 263K^MH^ was reminiscent yet not completely identical to that of 263K.

**Fig 6:**
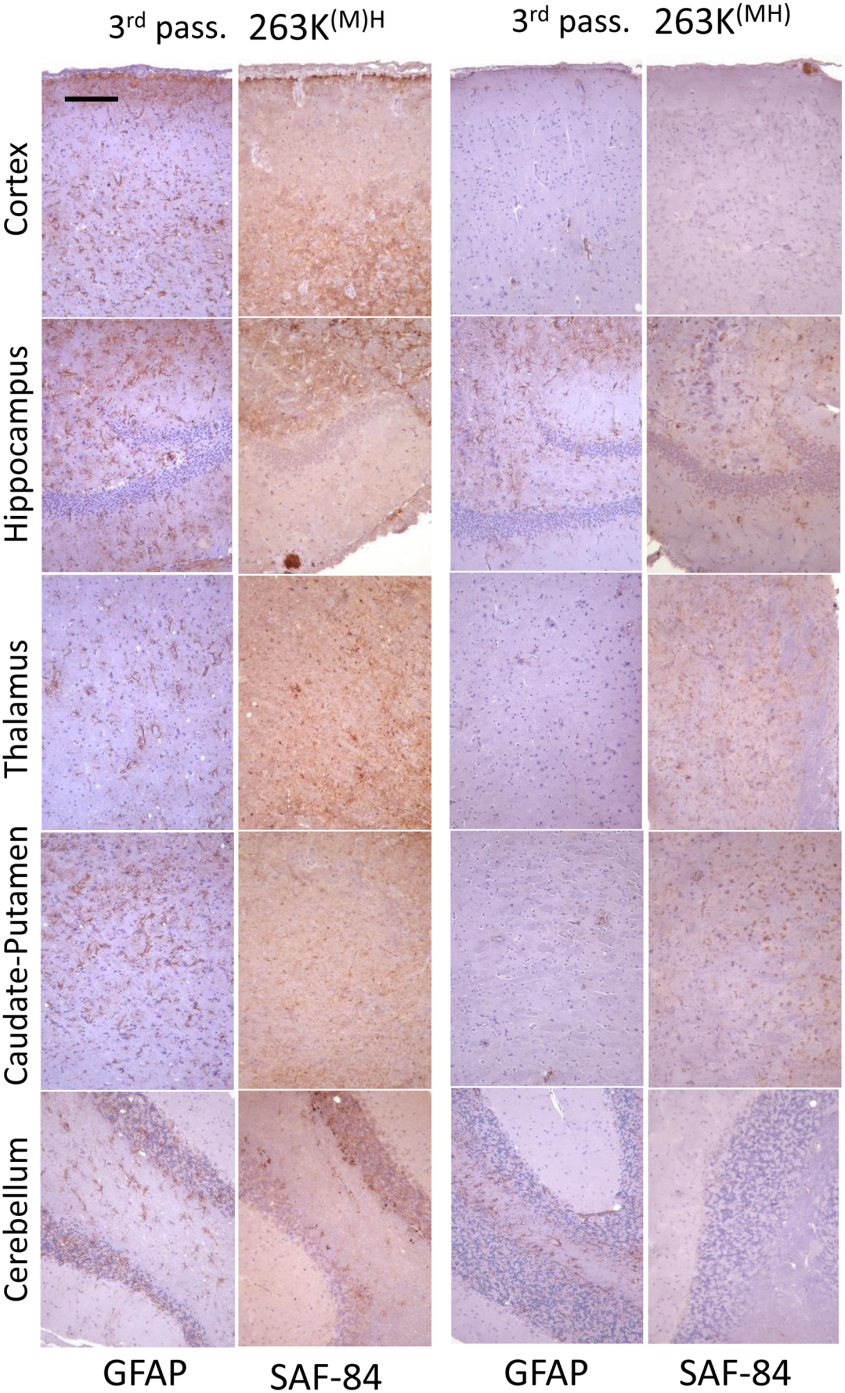
Histopathological analysis of brains from the 3^rd^ passage of 263K^(M)H^ and 263K^(MH)^. Representative images of the frontal cortex, hippocampus, thalamus, caudate-putamen, or cerebellum stained with anti-PrP SAF-84 or anti-GFAP antibodies as indicated. Scale bar = 100 μm for all images.

**Fig 7:**
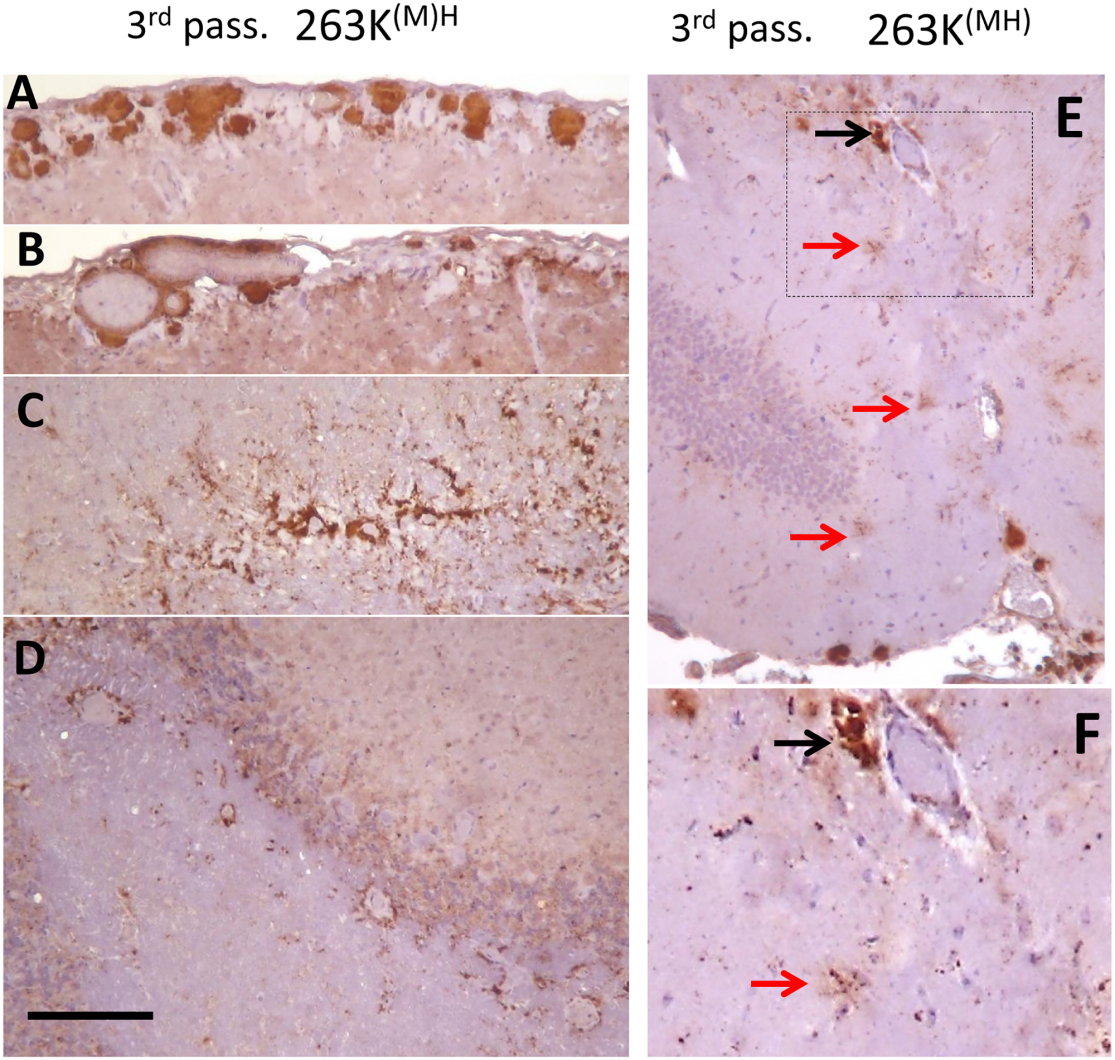
Histopathological analysis of PrP deposits in animals from the 3^rd^ passage of 263K^(M)H^ (A-D) and 263K^(MH)^ (E,F). Large pial deposits (A), deposits associated with leptomeningeal blood vessels (B), perivascular deposits in corpus callosum (C) and white matter cerebellum (D) were observed in 263K^(M)H^ group. The animals of 263K^(MH)^ showed perivascular (black arrow) and stellate (red arrows) deposits in hippocampus (E,F). Scale bar = 200 μm (A-E).

Histopathological analysis of the animals from the 3^rd^ passage of 263K^(M)H^ and 263K^(MH)^ revealed that both groups display characteristic features of prion disease including spongiform degeneration, reactive astrogliosis and PrP deposition (Fig 6, S8 Fig). Consistent with the clinical status and analysis of PrP^Sc^ by Western blot, 263K^(M)H^ group showed much more pronounced histopathological changes relative to the 263K^(MH)^ group. In 263K^(M)H^ group, significant spongiform changes, reactive astrogliosis and PrP deposition were observed throughout the brain (Fig 6). In 263K^(MH)^ group, the reactive astrogliosis, spongiform degeneration and PrP deposition were very mild across all brain regions (Fig 6, S9 Fig). With respect to the pattern of PrP deposition, unique features were found in both the 263K^(M)H^ and 263K^(MH)^ groups that were not seen in 263K^MH^ or 263K groups. In addition to dot-like and diffuse/synaptic immunoreactivity, 263K^(M)H^ group was characterized by numerous large pial deposits, deposits associated with leptomeningeal blood vessels, and intense perivascular deposits (Fig 7A–7D). The animals of 263K^(MH)^ showed predominantly dot-like deposits and some stellate deposits in hippocampus as well as perivascular deposits and smaller plaques (Fig 7E and 7F). Diffuse/synaptic immunoreactivity in 263K^(MH)^ group was much lower than in 263K^(M)H^ group. In both 263K^(M)H^ and 263K^(MH)^ groups, the cerebellum was less affected in comparison to 263K group. In summary, neuropathological analysis illustrated considerable changes in strain-specific characteristics in both 263K^(M)H^ and 263K^(MH)^ groups in comparison to 263K^MH^ or 263K groups.

## Discussion

The current study revealed that prion replication environment and specifically cellular RNAs play an important role in determining the fate of prion strain adaptation. We found that depletion of RNA in replication reactions changed the rate of strain adaptation and the disease phenotype upon serial passaging of PMCAb-derived material in animals. Serial passaging of 263K propagated under normal conditions in mouse and then hamster substrates (designated as 263K^MH^) resulted in a disease phenotype similar but not entirely identical to the original 263K. We do not know whether authentic 263K will emerge upon further serial transmission of 263K^MH^. Surprisingly, 263K propagated first in RNA-depleted mouse substrate and then normal hamster substrate (designated as 263K^(M)H^) resulted in a new disease phenotype. This disease phenotype was characterized by a longer incubation time and clinical duration of disease relative to the original 263K or 263K^MH^ group and altered neuropathological features. We do not know whether the incubation time of 263K^(M)H^ will shorten upon further serial transmission. 263K propagated first in RNA-depleted mouse and then RNA-depleted hamster substrates (designated as 263K^(MH)^) failed to produce clinical diseases for three serial passages despite persistent replication of PrP^Sc^ during serial transmission. Analysis of the PK-digestion patterns, glycoform ratios and GdnHCl-induced denaturation profiles revealed structural differences in PrP^Sc^ from 263K^MH^, 263K^(M)H^ and 263K^(MH)^ groups. In a manner similar to 263K, the internal sites of PK cleavage were exposed in 263K^MH^ PrP^Sc^ only after exposure to 3M GdnHCl. In contrast to 263K^MH^, the internal PK-cleavage sites were well-accessible in 263K^(MH)^ and mildly accessible in 263K^(M)H^ even under nondenaturing conditions (S5 Fig). In summary, three different outcomes of prion transmission were observed in animals depending on the presence or absence of RNA in PMCAb reactions.

Animals of all three groups 263K^MH^, 263K^(M)H^ and 263K^(MH)^ were asymptomatic in the first passage. Prior to inoculation into hamsters, 263K^MH^, 263K^(M)H^ and 263K^(MH)^ were amplified in serial PMCAb using hamster substrates. Therefore, the lack of clinical disease in the first passage cannot be attributed to the differences in the amino acid sequences of the PMCAb-derived inocula and PrP^C^ of the host. Instead, the lack of the disease could be in part due to a decline of prion-specific infectivity during serial PMCAb. Indeed, previous studies reported that the specific prion infectivity of 263K declines gradually during serial PMCA [37]. Nevertheless, it is difficult to attribute three asymptomatic serial passages of 263K^(MH)^ solely to a low specific infectivity of the PMCAb-derived products. In contrast to 263^MH^ and 263K^(M)H^ groups, in 263K^(MH)^ group the amounts of PrP^Sc^ increased very slowly during serial transmission. In fact, in the 2^nd^ passage most of the animals of 263^MH^ and 263K^(M)H^ groups showed considerable increase in the intensity of PrP^Sc^ signal relative to the signal intensity of the corresponding inocula, whereas the signal intensity for most of the animals of 263K^(MH)^ group was the same as in corresponding inoculum (Fig 2B). This could be due to intrinsically slow replication rate of 263K^(MH)^, its fast clearance, or both. Consistent with this conclusion, brain-derived 263K^(MH)^ was found to be considerably less resistant to proteolytic digestion at high concentrations of PK in comparison to the brain-derived 263^MH^ or 263K^(M)H^.

The hamster-adapted prion strain 263K used in the current study originated from the natural pool of scrapie that was isolated from the Cheviot breed in 1950 [38]. Since then, the 263K’s ancestor was transmitted through mice and rats before it was finally stabilized to hamsters after multiple serial passages [39]. Considering this history of interspecies passages, it is not surprising that 263K possesses certain level of plasticity and is able of overcoming a hamster-to-mouse species barrier. While 263K was initially regarded as nonpathogenic for mice as it failed to produce clinical disease in the first passage [40], subsequent studies documented slow or silent replication of 263K in mice that could lead to a clinical disease upon serial transmission [41–43]. Moreover, transgenic mice that overexpress mouse PrP^C^ (Tg20) developed clinical diseases in the first passage upon transmission of Sc237, the hamster-adapted strain of the same origin as 263K [34]. The ability of 263K PrP^Sc^ to recruit mouse PrP^C^ and convert it into PrP^Sc^ pathogenic to mice was illustrated further using *in vitro* experiments that employed PMCA [33]. Notably, the prion diseases observed in mice upon inoculation of PMCA-derived PrP^Sc^ seeded with 263K and amplified in mouse substrate showed unique disease phenotype [33]. Remarkably, other studies demonstrated that mice infected directly with hamster Sc237 PrP^Sc^ or PrP^Sc^ obtained upon replication of Sc237 in PMCA with mouse substrate produced different disease phenotypes upon serial transmission [34]. Those studies suggested that prion replication environment, whether it is *in vitro* environment of PMCA or environment of cellular sites of prion replication in a brain, is an important factor that determines disease phenotype upon cross-species transmission.

What is the role of RNA in prion replication? In previous studies, cellular and synthetic RNAs were shown to stimulate replication of prions *in vitro* [4,21]. The degree of the stimulating effect was found to be species- and strain-dependent [25,30,31]. While RNAs strongly facilitated replication of all hamster strains examined, the effect on replication of mouse strains was considerably less pronounced and strain-dependent [25,30,31]. Considering that the hamster strains are predominantly di-glycosylated, whereas the glycosylation statuses of mouse strains are variable, one can speculate that the species- and strain-dependency of the RNA effect could be due to differences in pattern or density of carbohydrate epitopes on PrP^Sc^ surface [44]. It is not clear whether RNAs assist prion replication *in vivo*. On one hand, convincing evidence have been presented that prion-specific polynucleotides are lacking in PrP^Sc^ particles isolated from Sc237-infected animals, the hamster-adapted strain of the same origin as 263K [45]. On the other hand, RNA molecules were found to co-localize with large extracellular PrP^Sc^ aggregates in hamsters infected with Sc237 [29]. Moreover, synthetic homopolymeric polynucleotides of sizes above 200 bases were found to stimulate conversion *in vitro* and form nuclease-resistant complexes with PrP molecules during PMCA reactions [29,46]. The detailed molecular mechanism behind the effects of RNA on prion replication is not known. Yet, it is reasonable to conclude that at least *in vitro* RNA provides favorable biochemical environment for prion replication. As suggested by previous studies, it is highly unlikely that the stimulating effects could be attributed to specific RNA sequences [4,46]. Instead, it appears that the polyanionic nature of RNA and, perhaps, its unique conformational features are important.

Application of PMCAb for examining the contribution of biochemical environment on fate of prion stain adaptation could be regarded as a main limitation of the current work. At the same time, the experimental design that involves PMCAb provides an opportunity for probing the hypothesis, which otherwise would be difficult, if not impossible, to test. Based on the results presented in the current work, one can speculate that accessibility of the cellular sites of prion replication to RNAs could be one of the factors that contribute to determining the fate of prion strain adaptation upon cross-species transmission. Among other factors that control the outcomes of the cross-species transmission are the transmission route and involvement of secondary lymphoid tissues in prion replication [47]. While it is difficult to fully disentangle strain adaptation *in vivo* from the adaptation that occurs during PMCAb reactions, the results of our previous studies helped to assess the contribution of the PMCAb technique itself to the apparent strain adaptation [48]. In those experiments, 10^3^-fold diluted 263K brain material was subjected to 24 serial PMCAb rounds with 1:10 dilution between rounds using only hamster NBH as a substrate. The resulting PMCAb-derived 263K was produced after an equal number of PMCAb rounds and dilution fold as in the current study. PMCAb-derived 263K was found to induce prion diseases in hamsters with 263K-specific disease phenotype an incubation time to the terminal stage 106±12 days postinoculation, which was longer than 82±2 days observed in animals inoculated with the equivalent amounts of brain-derived 263K. This experiment is consistent with the previous data that amplification of 263K in PMCAb in hamster substrate reduces specific prion infectivity, yet does not alter the disease phenotype [37]. Nevertheless, the fact that three serial passages were required for 263K^MH^ to exhibit the disease phenotype similar to 263K suggests that additional strain adaptation was taking place in hamsters during serial transmission.

The results of the current study can be discussed within two broad hypotheses—the cloud and deformed templating hypotheses, which are not mutually exclusive. According to the cloud hypothesis, the populations of PrP^Sc^ particles are intrinsically heterogeneous within individual strains or isolates due to spontaneous conformational mutations. PrP^Sc^ populations might consist of a major and a number of minor structural variants [49,50]. Upon a cross-species transmission, a minor PrP^Sc^ variant might become predominant in a new host due to changes in selection criteria and give rise to a new disease phenotype. If the cloud hypothesis accounts for the changes in fate of prion adaptation observed in the current studies, the current results suggest that altering a biochemical environment of prions replication *in vitro* gives a selective advantage to one of preexisting minor PrP^Sc^ variants resulting in an altered disease phenotype. The deformed templating model postulates that a change in replication environment plays an active role in generating new PrP^Sc^ variants, in addition to its role in imposing a new selective pressure [51,52]. PrP^Sc^ templates that do not fit well to a new environment still can seed altered PrP^Sc^ structural variants via deformed templating. While the majority of the newly generated variants might not replicate efficiently in altered environment, a variant that fits well to the new environment will eventually emerge through multiple trial-and-error seeding events. If one assumes that the deformed templating is behind observed effects, then 263K^(M)H^ and 263K^(MH)^ should be considered as two new variants that emerged *de novo* in RNA-depleted environment of PMCAb. It would be challenging to document what mechanism takes place, as it requires experimental testing of whether a minor PrP^Sc^ variant associated with a new disease phenotype pre-existed in the original prion isolate or strain. Nevertheless, our previous work demonstrated that PMCAb replication of 263K in RNA-depleted hamster substrate gave rise to new PrP^Sc^ variants that were absent in the original brain-derived 263K seeds [32]. Moreover, deformed templating mechanism describes well the evolution of prion strains of synthetic origin that were induced by recombinant PrP fibrils in animals despite fundamental structural differences between recombinant PrP fibrils and authentic PrP^Sc^ [53–56].

Regardless of which hypothesis is correct, the current work highlights a new important role of cofactor environment in prion cross-species transmission and adaptation. How changes in replication environment, and specifically RNA-depletion, can affect the fate of a prion strain? Previously, we suggested that adequate replication environment is necessary for insuring high fidelity of prion strain replication [32]. If this is true, RNA depletion during propagation of RNA-dependent strains could be compensated in part by other cellular polyanions creating heterogeneous replication environments and boosting diversity of the PrP^Sc^ variants. The current results support this mechanism, which is speculative at present time. Notably, in previous studies three mouse strains maintained their highly infectious and pathogenic state upon replication in PMCA in the presence of a lipid (phosphatidylethanolamine) as a sole cofactor, yet lost their original strain-specific features and converged into a single new strain [23]. Other studies reported transient changes in strain-specific clinical signs of the disease upon serial passaging of RML PrP^Sc^ that was subjected to replication in PMCA under RNA-depleted conditions [31]. In a second passage, the clinical signs of the disease reversed to the original RML-specific signs suggesting that the original RML variant overcompeted the new variant that emerged under the RNA-depleted conditions [31]. In summary, the current and previous studies suggest that maintaining adequate replication environment might be essential for maintaining prion strainness.

Recent study generated several novel prion strains by propagating chronic wasting disease prion isolates in PMCA that utilized recombinant bank vole PrP as a substrate and PrP knock-out mouse brain homogenate as a source of cofactors, and then replacing mouse brain homogenate with different cofactors of polyanionic nature [57]. Remarkably, the same set of PrP^Sc^ structures were generated in non-seeded or spontaneous PMCA reactions conducted only in the presence of recombinant bank vole PrP substrate and polyanionic cofactors [57]. These data suggest that cofactors might confine spontaneous PrP misfolding pathways *in vitro* while guiding it toward limited set of PrP^Sc^ structures that give rise to new prion strains upon transmission in animals. The current study tested the effect of RNA during cross-species adaptation and suggests that RNAs might be important for ensuring a high fidelity of prion replication.

In addition to cellular cofactors, what other parameters might be involved in determining the fate of prion cross-species transmission and strain adaptation? N-linked glycans represent the source of enormous diversity with respect to their composition and structure, yet their role in prion pathogenesis remains largely unknown. Our recent work revealed that PrP^C^ sialoglycoforms are recruited into PrP^Sc^ selectively in a strain-specific manner [58]. Based on 2D analysis of glycosylation of individual strains, we proposed that individual strain-specific structures of PrP^Sc^ govern selection of PrP^C^ sialoglycoforms that can be accommodated within individual structures producing a strain-specific pattern of carbohydrate epitopes on PrP^Sc^ surface [44,59]. In addition to a strain-specific structure, the pattern of carbohydrate epitopes is likely to be shaped by a host due to species-specific differences in the spectrum of N-linked glycans synthetized by different hosts. On one hand, transmission to a new host is likely to change carbohydrate patterns due to changes in PrP^Sc^ structure and exposure to a new pool of N-linked glycans in a new host. On the other hand, new pool of N-linked glycans might also play a role in selection of minor structural PrP^Sc^ variants in a new host. An interplay between the effect of PrP^Sc^ structure on selection of sialoglycoforms and conversely the effect of an altered pool of N-linked glycans on selection of PrP^Sc^ structural variants might explain the fact that stabilization of a new strain phenotype sometimes requires multiple serial passaging. Nevertheless, the role of N-linked glycans in determining the fate of prion strain adaptation has yet to be explored.

## Materials and methods

### Ethics statement

This study was carried out in strict accordance with the recommendations in the Guide for the Care and Use of Laboratory Animals of the National Institutes of Health. The animal protocol was approved by the Institutional Animal Care and Use Committee of the University of Maryland, Baltimore (Assurance Number A32000-01; Permit Number: 0215002).

### Animal bioassay

Four to five week old Golden Syrian hamsters (all males, Harlan Laboratories, Indianapolis, IN) were inoculated intracranically (IC) into the left hemisphere, ~3 mm to the left of the midline and ~3 mm anterior to a line drawn between the ears under 2% isoflurane anesthesia. Each animal received 50 μl of PMCAb-derived materials diluted 10-fold in 1% BSA/PBS. After inoculation, hamsters were observed daily for disease using a ‘blind’ scoring protocol. Animals from the first passage did not develop any clinical symptoms and were euthanized at 518 days post inoculation by asphyxiation with CO_2_ (Table 1). For the second and third passages, 10% brain homogenates (BH) in PBS were dispersed by 30 sec of sonication immediately before inoculation. Each hamster received 50 μl of 10% BH inoculum IC under 2% isoflurane anesthesia and was observed daily for disease using a ‘blind’ scoring protocol. Animals that developed clinical signs were sacrificed at the terminal stages of the diseases as indicated in Table 1, whereas animals that did not develop clinical signs were sacrificed at 503 days or 614 days post inoculation for the second and third passages, respectively (Table 1).

### Protein Misfolding Cyclic Amplification with beads

PMCAb procedure has been described in detail elsewhere [60]. Briefly, healthy hamsters or mice were euthanized and immediately perfused with PBS, pH 7.4, supplemented with 5 mM EDTA. 10% brain homogenate (w/v) was prepared using ice-cold conversion buffer (Ca^2+-^free and Mg^2+^-free PBS, pH 7.5, 0.15 M NaCl, 1.0% Triton supplemented with 1 tablet of Complete protease inhibitors cocktail (cat # 1836145, Roche) per 50 mL of buffer) and glass/Teflon homogenizers attached to a cordless 12 V compact drill (Ryobi). The brains were ground at low speed until homogeneous, and then five additional strokes completed the homogenization. The resulting 10% normal brain homogenate (NBH) was used as the substrate in PMCAb reactions.

To produce RNA-depleted NBH, pre-cleared 10% brain homogenate was incubated with 100 *μ*g/mL of RNase A (cat # R-4875, Sigma-Aldrich) for 1 hour at 37°C under gentle rotation prior to its use as substrate in PMCAb. RNA-depletion in RNase-treated NBH was confirmed by agarose gel as previously described [25]. To prepare seeds, 10% 263K brain homogenates in PBS were diluted 10-fold in the conversion buffer, and 10 *μ*L of the dilution was used to seed amplification in 90 *μ*L of fresh substrate. Samples in 0.2 mL thin-wall PCR tubes (Fisher, Cat. No. 14230205) supplemented with 3 Teflon beads (McMaster-Carr, Robbinsville, NJ) were placed in a floating rack inside a Misonix S-4000 microplate horn (Qsonica LLC, Newtown, CT) filled with 350 mL of water. Two coils of rubber tubing attached to a circulating water bath were installed to maintain 37 °C inside the sonicator chamber. The standard sonication program consisted of 30 s sonication pulses delivered at 50% power efficiency applied every 30 min during a 24 h period, consisting each round of 48 cycles. 10-fold dilutions were used between serial rounds in both mouse and hamster substrates.

### Proteinase K digestion

To analyze PMCAb products, 10 μl of each sample were supplemented with 2.5 μl SDS and 2.5 μl PK (cat. #P8107S, New England BioLabs, Ipswich, MA), to a final concentration of SDS and PK of 0.25% and 50 μg/ml respectively, followed by incubation at 37°C for 1 hour. The digestion was terminated by addition of SDS-sample buffer and boiling for 10 min. Samples were loaded onto NuPAGE 12% BisTris gels, transferred to PVDF membrane, and stained with 3F4, D18 or SAF-84 primary antibody for detecting PrP^Sc^ as indicated.

To analyze scrapie BH, an aliquot of 10% brain homogenate was mixed with an equal volume of 4% sarcosyl in PBS, supplemented with 50 mM Tris, pH 7.5, and digested with 20 μg/ml PK (New England BioLabs) for 1 hour at 37°C with 1000 rpm shaking. The digestion was terminated by addition of SDS-sample buffer and boiled for 10 min and loaded onto NuPAGE 12% BisTris gels. After transfer to PVDF membrane, PrP^Sc^ was detected with 3F4 or SAF-84 primary antibody as indicated.

For the analysis of PK-resistance profile, 10% BHs of animals from the 3^rd^ passage were diluted 10-fold into 4% sarkosyl in PBS and sonicated for 30 sec, then supplemented with equal volume of 100 mM Tris, pH 7.5. The samples were centrifuged 5 min at 16,000 g to remove debris. The supernatant was digested with PK (New England BioLabs) at increasing concentrations (2, 10, 50, 250, 1,000, and 2,000 μg/ml) for 30 min at 37°C under shaking. The digestion was terminated by addition of SDS-sample buffer and heating for 10 min in a boiling water bath. Analysis of GdnHCl-induced denaturation was performed as previously described [54].

Western blot signal intensity was digitized for densitometry analysis using AlphaView software (ProteinSimple, San Jose, CA). Target bands were selected using a uniform rectangular sampling area that encompassed the band of interest. Background optical density of an equal area from the same blot was determined and subsequently subtracted from the density of the bands. The values were normalized, with value at 2 μg/ml PK taken as 100%. Three independent brains were analyzed for each sample type, for calculating mean and standard deviations. The plots were drawn in Microsoft Excel.

### Histopathological study

Histopathological studies were performed on three animals per group. Formalin fixed brain halves were divided at the midline. Right hemisphere was frozen, and left hemisphere was fixed in 10% neutral buffered formalin solution. Formalin-fixed hemispheres were paraffin embedded, sliced into 4 μm sections and processed for hematoxylin-eosin stain as well as for immunohistochemistry for PrP using the mouse monoclonal anti-PrP antibody SAF-84 (1:1000, Cayman Chemical, Ann Arbor, Michigan, USA) or anti-glial fibrillar acidic protein (GFAP; 1:3000, Dako, Glostrup, Denmark). Horse radish peroxidase-labeled goat anti-rabbit and anti-mouse antibody (KPL, Milford, MA) were used as secondary antibody for GFAP (rabbit) and SAF-84 (mouse). Detection was performed using DAB Quanto chromogen and substrate (VWR, Radnor, PA). Brains were treated in formic acid (96%) prior to embedding in paraffin to deactivate prion infectivity. For detection of disease-associated PrP, we applied a pretreatment of 30 minutes hydrated autoclaving at 121°C followed by 5 minutes in 96% formic acid. As age-matched normal controls for the histopathology study, Golden Syrian hamsters of 660 days old were used. This age corresponds to the biological age of the oldest experimental group euthanized in the current work.

## Supporting information

S1 Fig Analysis of RNA content in RNA-depleted NBH. 10% NBH was treated with RNase A for 1 hour, then RNA content was analyzed using 1.2% Agarose gel and stained with ethidium bromide. Mock-digested 10% NBH is shown as a reference. DNA markers were used as running standards.
(PDF)

S2 Fig Histopathological analysis of spongiform in the brains from the 2^nd^ passage of 263K^MH^, 263K^(M)H^ or 263K^(MH)^. Representative images of the frontal cortex, hippocampus, thalamus, caudate-putamen, or cerebellum stained with hematoxylin and eosin. In contrast to the 263K^MH^ and 263K^(M)H^ groups, the 263K^(MH)^ animals showed very minor if any vacuolation. Scale bar in left upper image represents 100 μm for all images.
(PDF)

S3 Fig Lesion profile (left panel) and PrP immunopositivity score (right panel) in hamsters from the 2^nd^ passage of 263K^MH^, 263K^(M)H^ or 263K^(MH)^. The lesion profile was obtained by averaging the scores for spongiform change, neuronal loss and gliosis for three animals within each group. The PrP immunopositivity profile was obtained by averaging the scores for three animals within each group.
(PDF)

S4 Fig Histopathological analysis of spongiform changes using staining with hematoxylin and eosin (left panels) or PrP immunoreactivity using SAF-84 antibody (right panels) of aged-matched control group. Representative images of the frontal cortex, hippocampus, thalamus, caudate-putamen, or cerebellum in three animals from the aged-matched control group. Scale bar in left upper image represents 100 μm for all images.
(PDF)

S5 Fig Analysis of GdnHCl-induced denaturation profiles of PrP^Sc^. 1% BHs from 263K and 3d passages of 263K^MH^, 263K^(M)H^ and 263K^(MH)^ groups were incubated with increasing concentrations of GdnHCl from 0.4 to 4 M for 1 h, as indicated, then diluted out of GdnHCl, equilibrated for 1 h at room temperature and digested with 20 μg/ml PK. Undigested brain material exposed to 0.4 M GdnHCl is provided as a reference. Western blots were stained with SAF-84 antibody. Arrow indicates blind spots on Western blots that arose due to transfer of PK.
(PDF)

S6 Fig Histopathological analysis of spongiform in the brains from the 2^nd^ and 3^rd^ passages of 263K^MH^ and animals inoculated with 263K. Representative images of the frontal cortex, hippocampus, thalamus, caudate-putamen, or cerebellum stained with hematoxyilin and eosin. Scale bar = 100 μm for all images.
(PDF)

S7 Fig Immunostaining for PrP displayed the following types of PrP deposits: Diffuse/Synaptic fine deposits, intra- and perineuronal deposits, plaques and mini-plaques in the subependymal area, small granular accumulations on the ependyma, and amorphous plaque-like deposits in the subpial and subependymal regions. The scale bar in the left upper image represents 10 μm for all images.
(PDF)

S8 Fig Lesion profile (left panel) and PrP immunopositivity score (right panel) in hamsters from the 2^nd^ and 3^rd^ passages of 263K^MH^ and 263K groups. The lesion profile was obtained by averaging the scores for spongiform change, neuronal loss and gliosis for three animals within each group. The PrP immunopositivity profile was obtained by averaging the scores for three animals within each group.
(PDF)

S9 Fig Histopathological analysis of spongiform in the brains from the 3^rd^ passage of 263K^(M)H^ and 263K^(MH)^. Representative images of the frontal cortex, hippocampus, thalamus, caudate-putamen, or cerebellum stained with hematoxylin and eosin. Scale bar = 100 μm for all images.
(PDF)
