# Supplementary figures and images for: Prion replication environment defines the fate of prion strain adaptation
Source: PLoS Pathog. 2018 Jun 21;14(6):e1007093. doi: 10.1371/journal.ppat.1007093 (PMC6013019; doi:10.1371/journal.ppat.1007093)

Figure S1

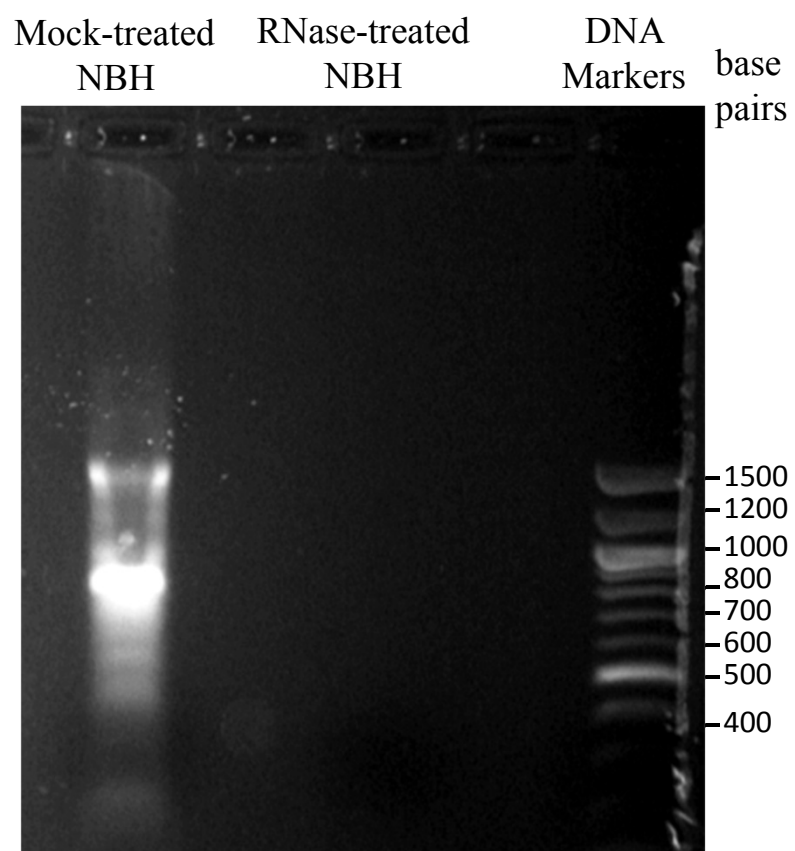

Supplement: S1 Fig — 10% NBH was treated with RNase A for 1 hour, then RNA content was analyzed using 1.2% Agarose gel and stained with ethidium bromide. Mock-digested 10% NBH is shown as a reference. DNA markers were used as running standards. (PDF) [file ppat.1007093.s001.pdf]

Figure S3

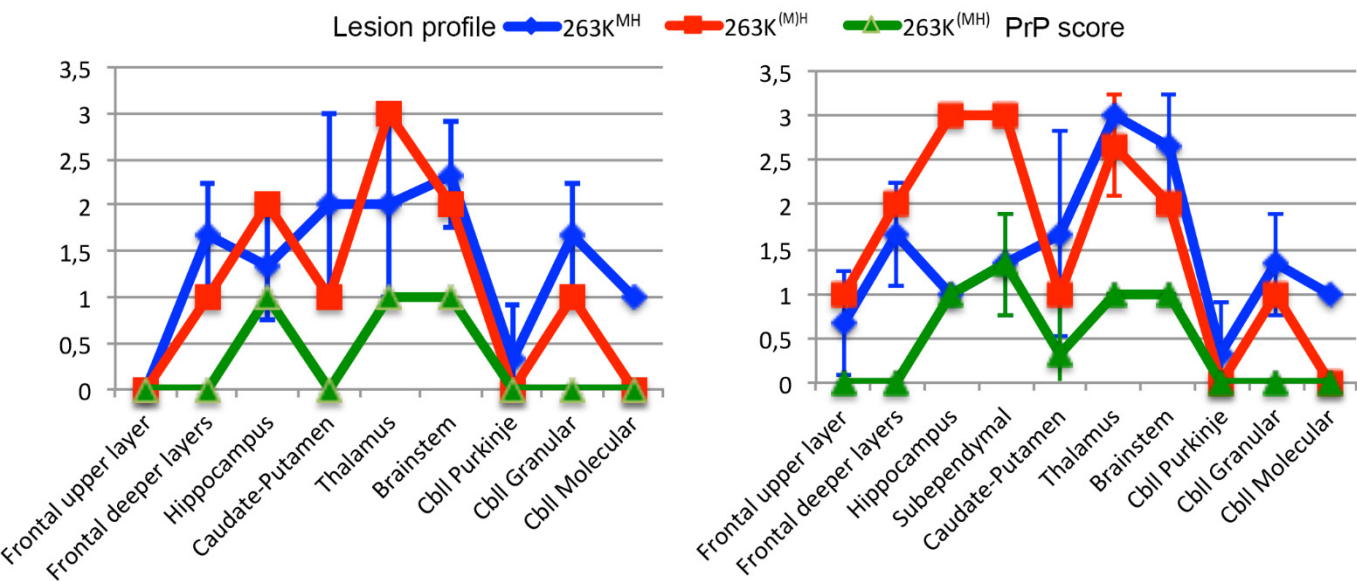

Supplement: S3 Fig — The lesion profile was obtained by averaging the scores for spongiform change, neuronal loss and gliosis for three animals within each group. The PrP immunopositivity profile was obtained by averaging the scores for three animals within each group. (PDF) [file ppat.1007093.s003.pdf]

Figure S4

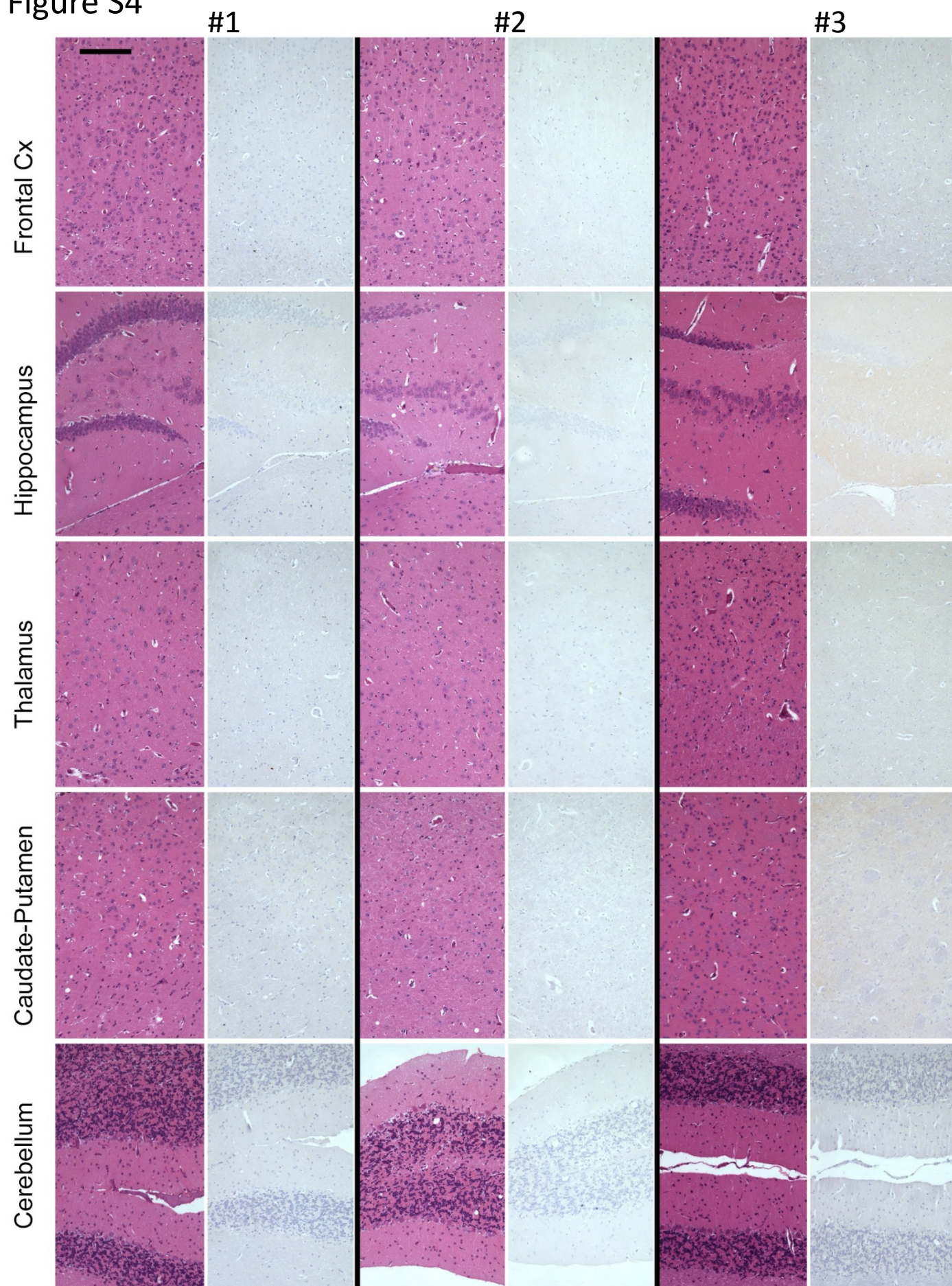

Supplement: S4 Fig — Representative images of the frontal cortex, hippocampus, thalamus, caudate-putamen, or cerebellum in three animals from the aged-matched control group. Scale bar in left upper image represents 100 μm for all images. (PDF) [file ppat.1007093.s004.pdf]

Figure S5

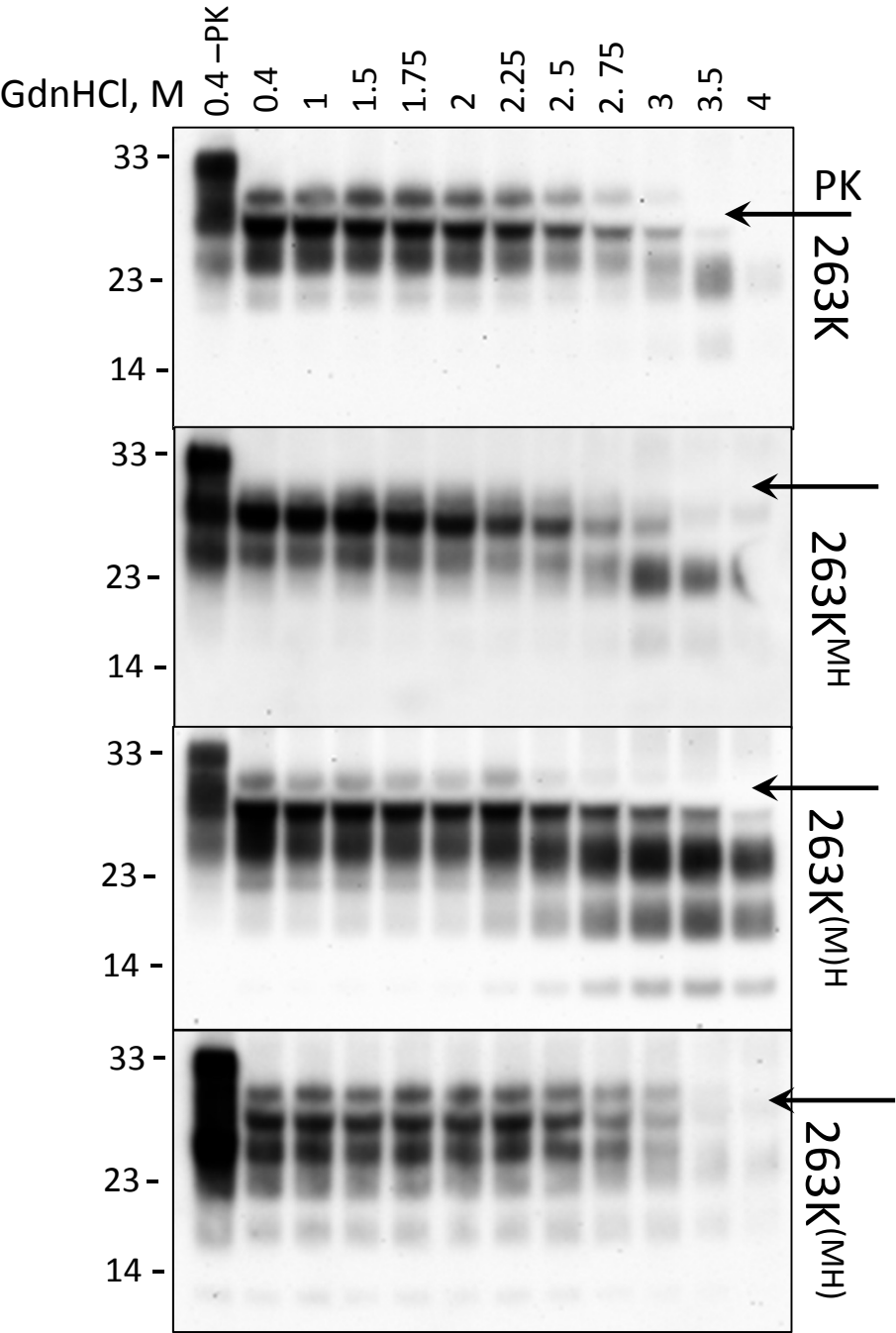

Supplement: S5 Fig — 1% BHs from 263K and 3d passages of 263KMH, 263K(M)H and 263K(MH) groups were incubated with increasing concentrations of GdnHCl from 0.4 to 4 M for 1 h, as indicated, then diluted out of GdnHCl, equilibrated for 1 h at room temperature and digested with 20 μg/ml PK. Undigested brain material exposed to 0.4 M GdnHCl is provided as a reference. Western blots were stained with SAF-84 antibody. Arrow indicates blind spots on Western blots that arose due to transfer of PK. (PDF) [file ppat.1007093.s005.pdf]

Figure S6

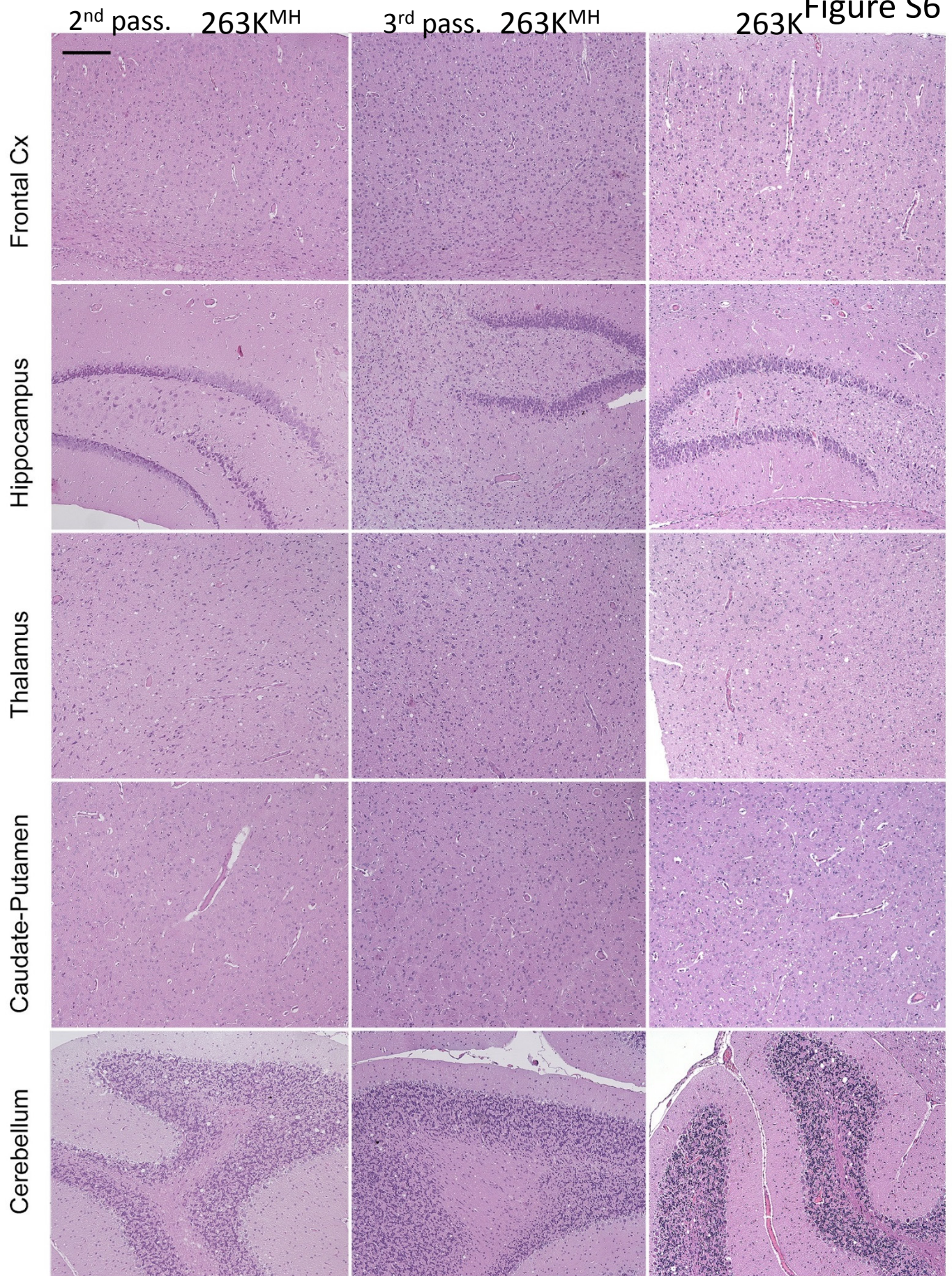

Supplement: S6 Fig — Representative images of the frontal cortex, hippocampus, thalamus, caudate-putamen, or cerebellum stained with hematoxyilin and eosin. Scale bar = 100 μm for all images. (PDF) [file ppat.1007093.s006.pdf]

Figure S8

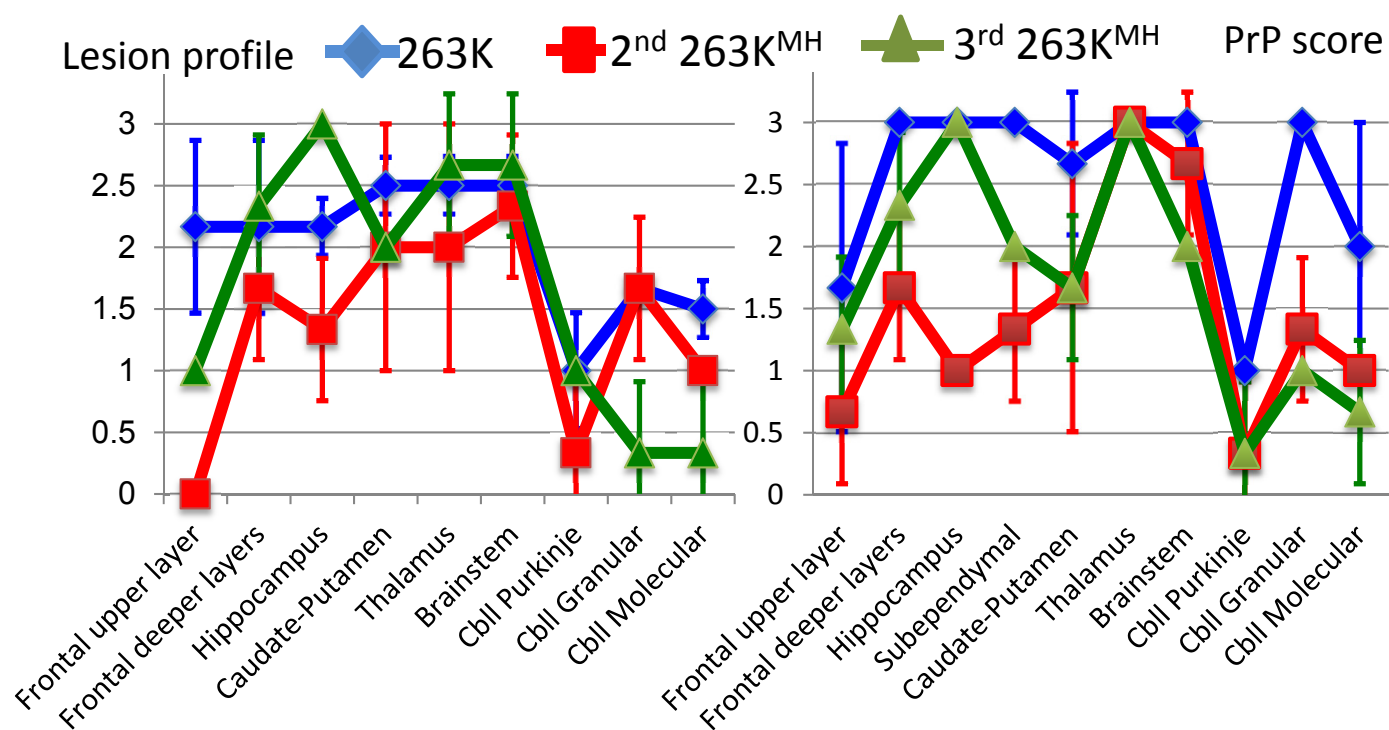

Supplement: S8 Fig — The lesion profile was obtained by averaging the scores for spongiform change, neuronal loss and gliosis for three animals within each group. The PrP immunopositivity profile was obtained by averaging the scores for three animals within each group. (PDF) [file ppat.1007093.s008.pdf]

Figure S9

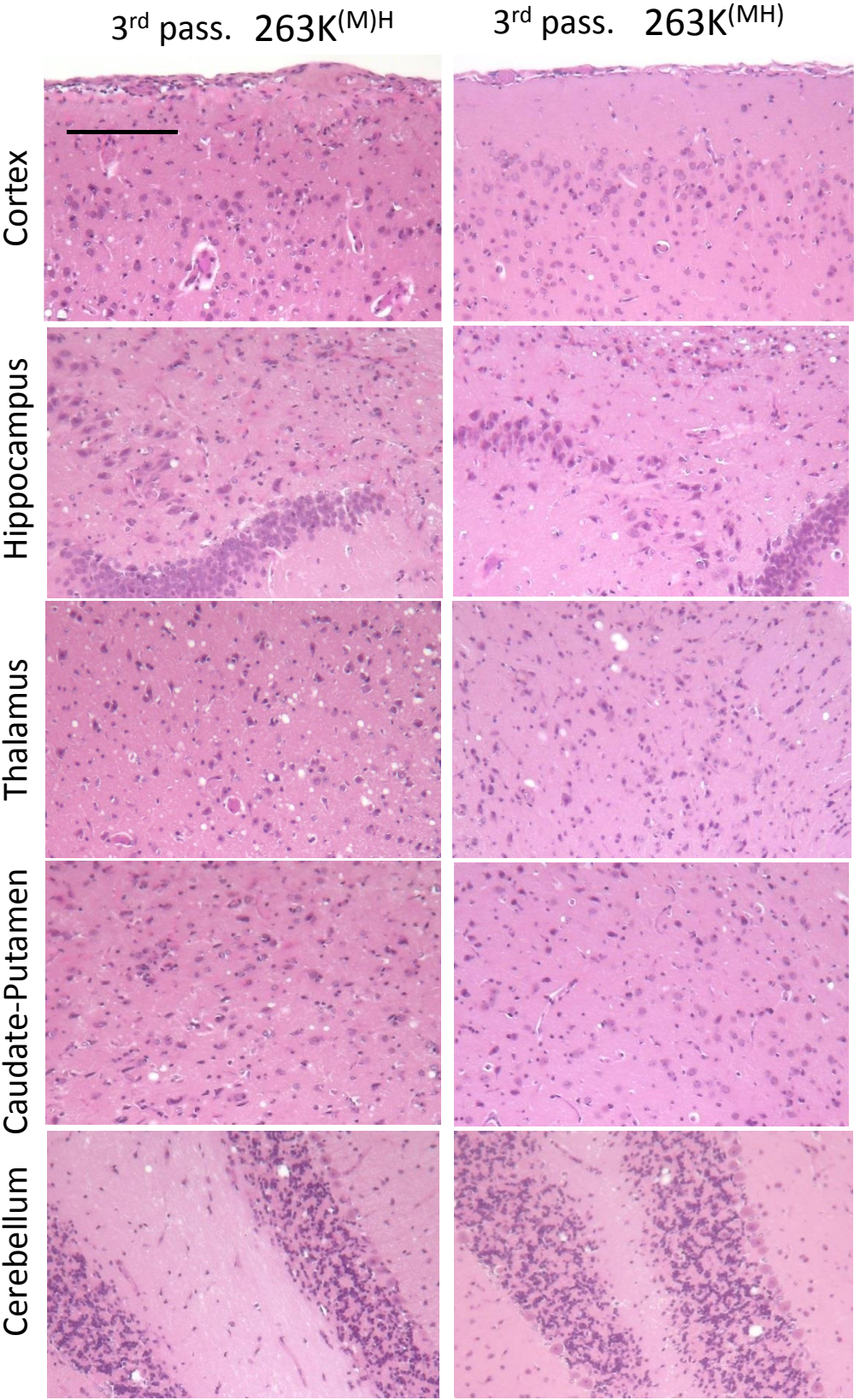

Supplement: S9 Fig — Representative images of the frontal cortex, hippocampus, thalamus, caudate-putamen, or cerebellum stained with hematoxylin and eosin. Scale bar = 100 μm for all images. (PDF) [file ppat.1007093.s009.pdf]
