# Supplementary material for: Prion replication environment defines the fate of prion strain adaptation
Source: PLoS Pathog. 2018 Jun 21;14(6):e1007093. doi: 10.1371/journal.ppat.1007093 (PMC6013019; doi:10.1371/journal.ppat.1007093)
Supplement: S2 Fig — Representative images of the frontal cortex, hippocampus, thalamus, caudate-putamen, or cerebellum stained with hematoxylin and eosin. In contrast to the 263KMH and 263K(M)H groups, the 263K(MH) animals showed very minor if any vacuolation. Scale bar in left upper image represents 100 μm for all images. (PDF) [file ppat.1007093.s002.pdf]

Figure S2

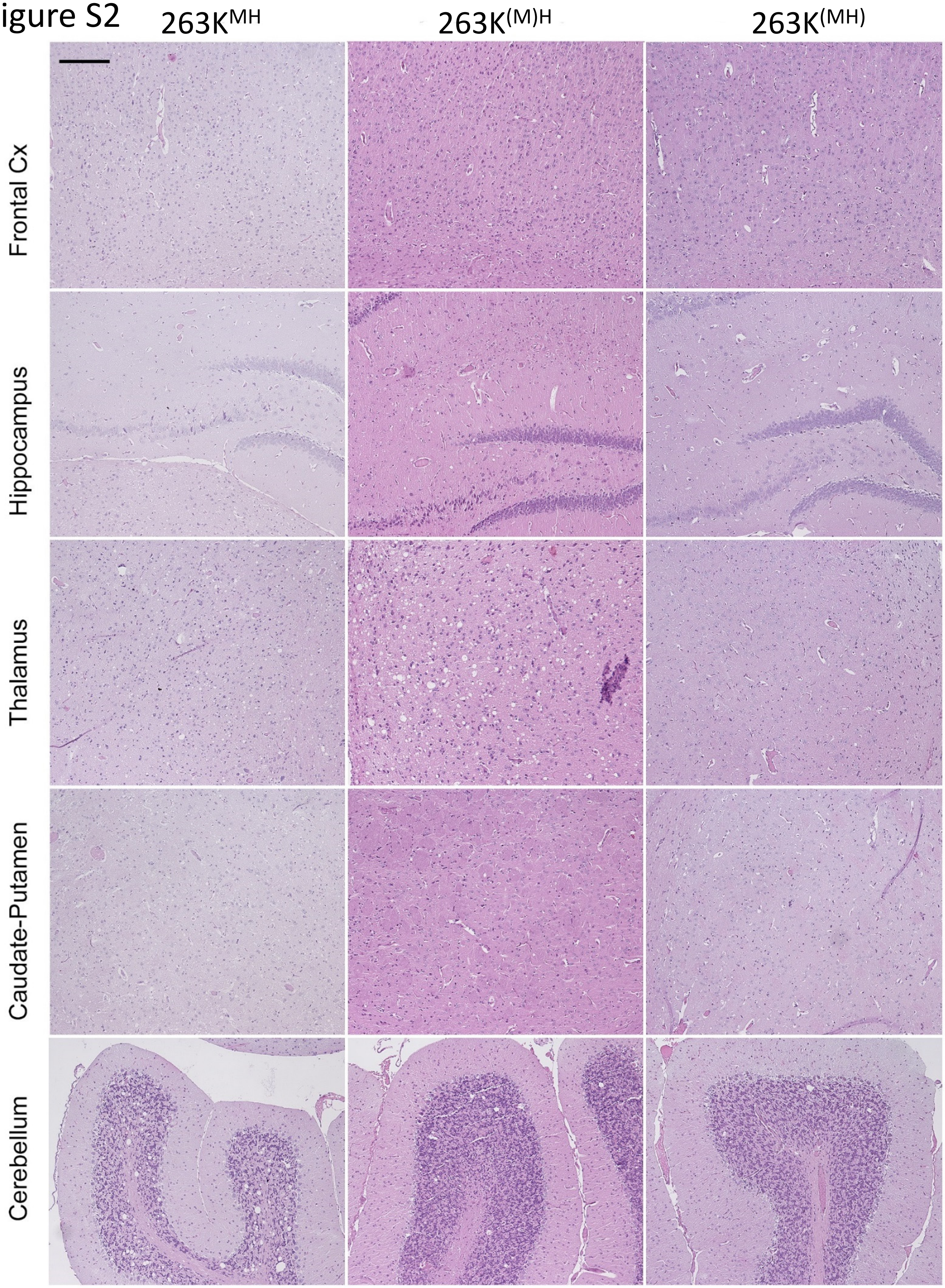
263K^MH
263K^(M)H
263K^(MH)
Frontal Cx
Hippocampus
Thalamus
Caudate-Putamen
Cerebellum
