# Supplementary material for: Prion replication environment defines the fate of prion strain adaptation
Source: PLoS Pathog. 2018 Jun 21;14(6):e1007093. doi: 10.1371/journal.ppat.1007093 (PMC6013019; doi:10.1371/journal.ppat.1007093)
Supplement: S7 Fig — The scale bar in the left upper image represents 10 μm for all images. (PDF) [file ppat.1007093.s007.pdf]

Figure S7

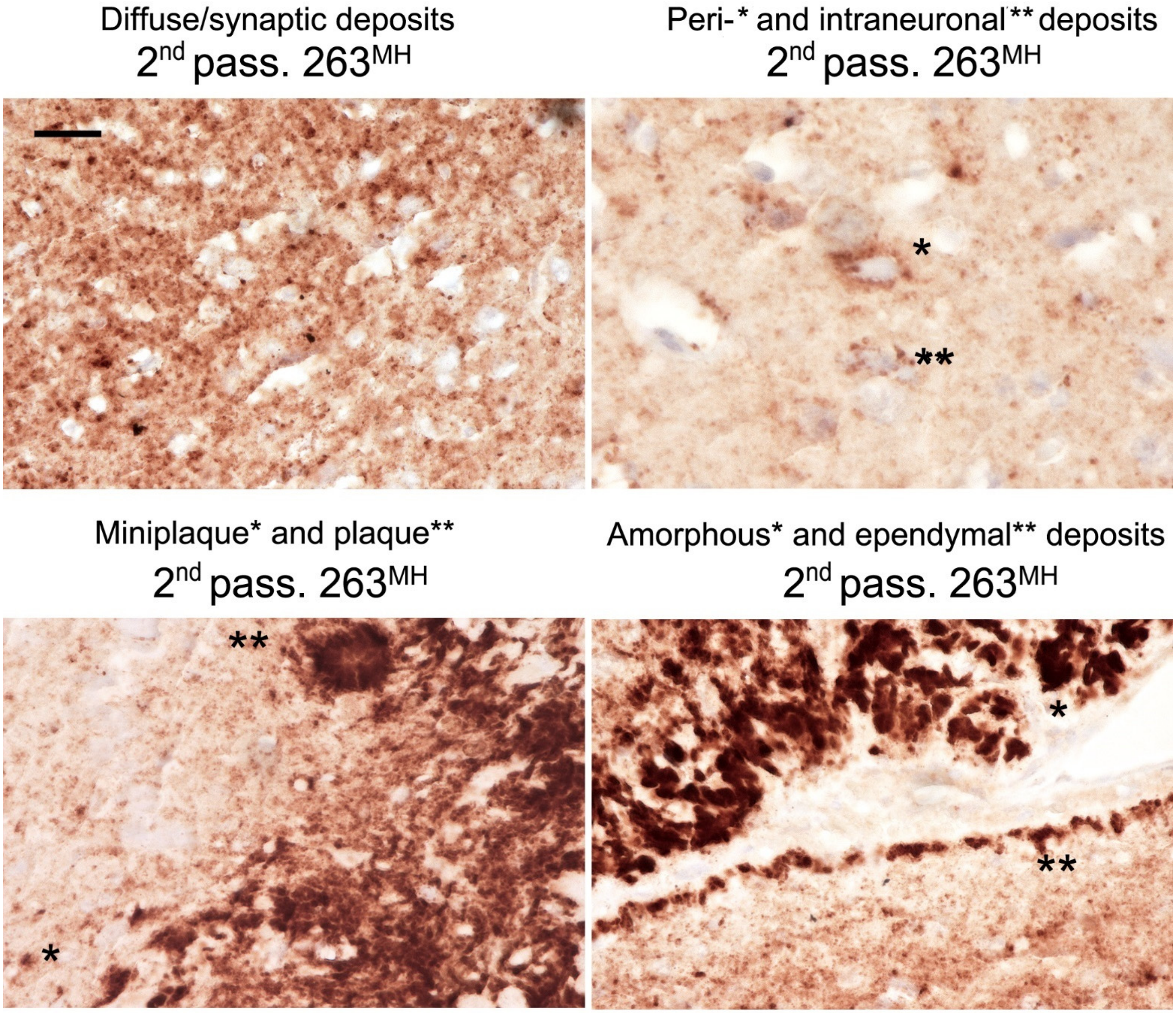

Diffuse/synaptic deposits
2nd pass. 263MH
Peri-* and intraneuronal** deposits
2nd pass. 263MH
*
**
Miniplaque* and plaque**
2nd pass. 263MH
**
*
Amorphous* and ependymal** deposits
2nd pass. 263MH
*
**
